# Host Cell Membrane Capture by the SARS-CoV-2 Spike Protein Fusion Intermediate

**DOI:** 10.1021/acscentsci.3c00158

**Published:** 2023-06-07

**Authors:** Rui Su, Jin Zeng, Tara C. Marcink, Matteo Porotto, Anne Moscona, Ben O’Shaughnessy

**Affiliations:** † Department of Chemical Engineering, Columbia University, New York, New York 10027, United States; ‡ Department of Pediatrics, Columbia University Vagelos College of Physicians & Surgeons, New York, New York 10032, United States; § Center for Host−Pathogen Interaction, Columbia University Vagelos College of Physicians & Surgeons, New York, New York 10032, United States; ∥ Department of Experimental Medicine, University of Campania “Luigi Vanvitelli”, 81100 Caserta, Italy; ⊥ Department of Microbiology & Immunology, Columbia University Vagelos College of Physicians & Surgeons, New York, New York 10032, United States; # Department of Physiology, Columbia University Vagelos College of Physicians & Surgeons, New York, New York 10032, United States

## Abstract

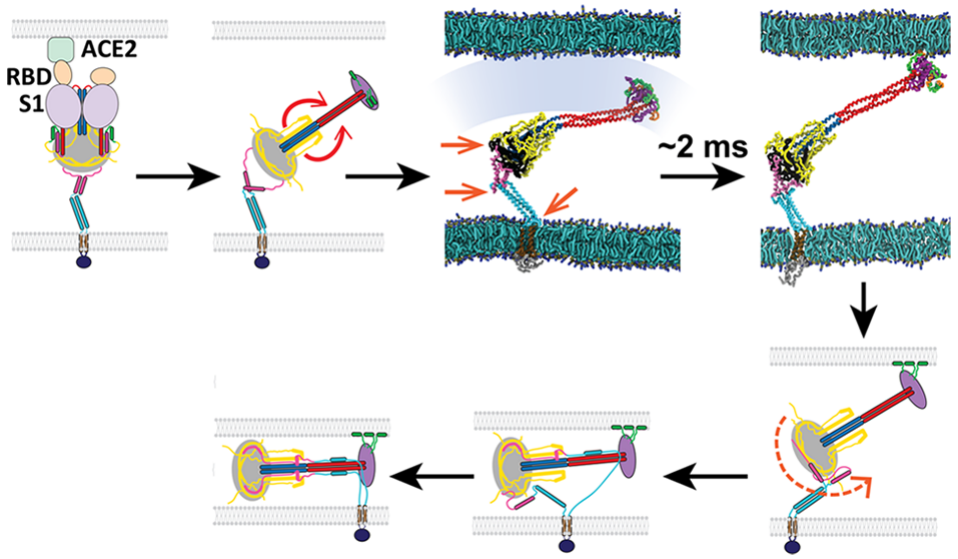

Cell entry by SARS-CoV-2 is accomplished by the S2 subunit of the spike S protein on the virion surface by capture of the host cell membrane and fusion with the viral envelope. Capture and fusion require the prefusion S2 to transit to its potent fusogenic form, the fusion intermediate (FI). However, the FI structure is unknown, detailed computational models of the FI are unavailable, and the mechanisms and timing of membrane capture and fusion are not established. Here, we constructed a full-length model of the SARS-CoV-2 FI by extrapolating from known SARS-CoV-2 pre- and postfusion structures. In atomistic and coarse-grained molecular dynamics simulations the FI was remarkably flexible and executed giant bending and extensional fluctuations due to three hinges in the C-terminal base. The simulated configurations and their giant fluctuations are quantitatively consistent with SARS-CoV-2 FI configurations measured recently using cryo-electron tomography. Simulations suggested a host cell membrane capture time of ∼2 ms. Isolated fusion peptide simulations identified an N-terminal helix that directed and maintained binding to the membrane but grossly underestimated the binding time, showing that the fusion peptide environment is radically altered when attached to its host fusion protein. The large configurational fluctuations of the FI generated a substantial exploration volume that aided capture of the target membrane, and may set the waiting time for fluctuation-triggered refolding of the FI that draws the viral envelope and host cell membrane together for fusion. These results describe the FI as machinery that uses massive configurational fluctuations for efficient membrane capture and suggest novel potential drug targets.

## Abstract 2

SARS-CoV-2 unleashes the spike fusion intermediate to enter cells. Simulations reveal 3 base hinges mediating giant fluctuations that enable target cell capture after ∼2 ms and may trigger refolding.

## Introduction

The COVID-19 global pandemic is this century’s third coronavirus epidemic following SARS-CoV in 2002 and Middle East respiratory syndrome coronavirus (MERS-CoV) in 2012, suggesting that coronaviruses will remain a global health threat for the foreseeable future. The responsible pathogen is the severe acute respiratory syndrome coronavirus 2 (SARS-CoV-2), a ∼100-nm-diameter betacoronavirus^1^ whose lipid envelope encloses a positive-sense single-stranded RNA genome complexed with the nucleocapsid protein. The lipid envelope houses the spike (S) glycoprotein, the envelope protein, and the membrane protein.^2^ The S protein is a trimeric class I fusion protein that catalyzes entry into susceptible cell types and has two subunits, S1 and S2.^3,4^ Following S1-mediated binding to host cell membrane Angiotensin-Converting Enzyme 2 (ACE2) receptors, S1 and S2 dissociate, releasing S2.

Entry is the job of the S2 subunit, by fusion of the viral envelope and host cell membrane. To become fusion competent, S2 must first undergo a major structural transition from its prefusion state to the potent, fusogenic form, the extended fusion intermediate (FI)^5,6^ which bears three N-terminal fusion peptides (FPs) that capture the host cell membrane. Subsequent refolding of the fusion intermediate into its postfusion configuration^7^ pulls the viral envelope and target membrane together for fusion and delivery of viral genomic material.^8^

The fusion intermediate is the unsheathed weapon of entry by SARS-CoV-2, but the mechanism and timescales of FI-mediated host cell membrane capture are unknown. Little is known about this critical machinery. While the prefusion and postfusion SARS-CoV-2 S protein structures are known from cryo-EM and X-ray crystallography,^3,7,9,10^ the FI structure is undetermined. Extended intermediate states of class I fusion proteins have generally proved experimentally elusive, likely due to their estimated sec–min lifetimes, far shorter than the pre- or postfusion lifetimes.^5,11,12^ Indeed, no class I fusion protein intermediate had been visualized until very recently, when the HIV-1 gp41 intermediate, the parainfluenza F intermediate, and the influenza HA2 intermediate^12−14^ were visualized with cryo-EM for the first time.

The spike protein is a target for vaccines, therapeutic antibodies, fusion-inhibitory peptides, and other antivirals. Most current recombinant neutralizing or vaccine-elicited antibodies to SARS-CoV-2 bind S1.^15−18^ Viral fusion inhibitors targeting S2^10,19^ and the fusion-executing subunits of other class I fusion proteins have been developed, including one FDA-approved drug against HIV.^20−22^ Importantly, recently emerged SARS-CoV-2 variants harboring spike protein mutations escaped from two S1-targeting antibodies, one of which has FDA emergency authorization, whereas the efficiency of S2-targeting peptide antivirals was unaffected.^23,24^ Moreover, S2 is conserved among coronaviruses.^25^ Thus, unveiling the mechanisms of the SARS-CoV-2 S2 fusion intermediate will be vital in the search for robust and pan-coronavirus antiviral drugs.

Detailed computational studies of the SARS-CoV-2 FI and the kinetics of FI-mediated membrane capture are unavailable, to our knowledge. However, the prefusion S protein was atomistically simulated,^26−28^ one study revealing a highly flexible prefusion structure due to three hinge-like regions, consistent with cryo-ET.^27^ Membrane binding by the isolated FP was simulated,^29−31^ including Ca^2+^-dependent binding which involved the conserved coronavirus LLF motif in the N-terminal FP helix.^29^ A coarse-grained model of the whole virion was developed.^32^ For other class I fusion proteins, a model structure of the Ebola GP2 extended intermediate was constructed,^33^ and local transitions of the influenza HA and HIV gp41 intermediates were modeled.^34,35^

Here, we constructed a full-length structural model of the SARS-CoV-2 FI by extrapolating from known SARS-CoV-2 pre- and postfusion structures. The model suggests that a “loaded spring” mechanism triggers the prefusion-to-FI transition, similar to that for influenza HA, which is thought triggered by folding of the B-loop into a helix.^36^ We studied membrane capture by the FI, using all-atom and MARTINI coarse-grained molecular dynamics simulations to access timescales up to a millisecond. Simulations showed the FI is highly flexible, subject to large orientational and extensional fluctuations due to three hinges in the C-terminal base closely related to the prefusion hinges.^27^ Quantitative comparison with our recently obtained cryo-electron tomograms of the SARS-CoV-2 FI supports these giant configurational fluctuations, which greatly increase the volume swept out by the FI. We suggest that this helps the FI capture target cell membrane. A critical N-terminal amphiphilic helix in the FP mediates membrane binding, but we find that FP-only simulations severely mispresent the kinetics, as membrane capture is far slower in the native structure with the FP attached to the FI. Our coarse-grained simulations suggest that FI-mediated membrane capture requires ∼2 ms, a critical step on the pathway to fusion. In addition to facilitating membrane capture, we propose that large FI fluctuations set the timing of fluctuation-triggered FI refolding on the pathway to membrane fusion. Our work identifies several novel potential drug targets.

## Results

### Model of the SARS-CoV-2 Spike Protein Fusion Intermediate

**Figure 1:**
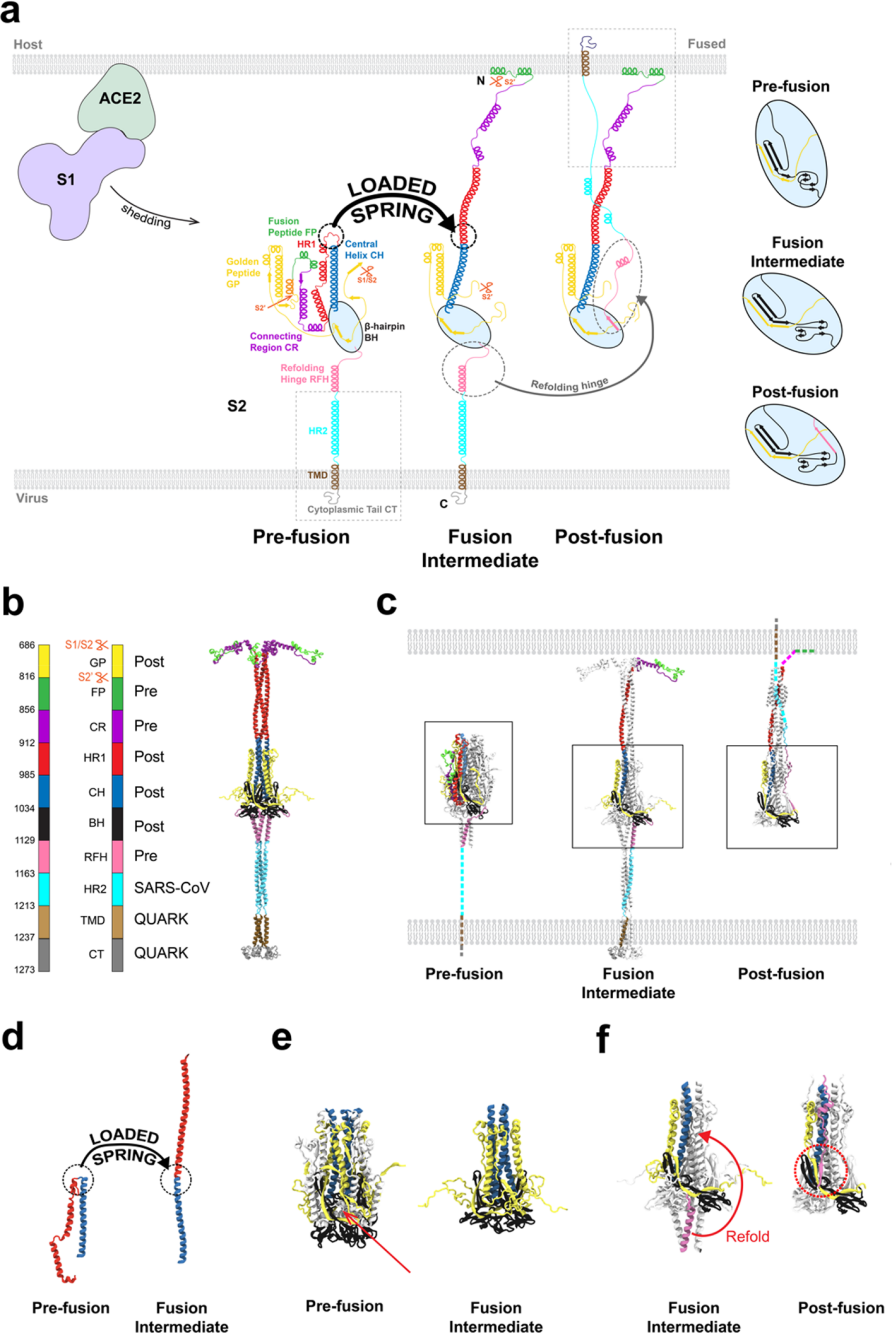
Model of the SARS-CoV-2 spike protein fusion intermediate. (a) Model of the fusion intermediate (FI) of the SARS-CoV-2 spike (S) protein, schematic. One protomer of the S trimer is shown. Schematics of the pre- and postfusion S2 states from the known structures, other than unknown regions (boxed). Details of BH and adjacent domains, at right. Following dissociation of S2 from S1, the unstructured prefusion HR1 loops become helical (loaded spring release), giving the HR1-CH backbone that thrusts the FPs toward the host cell membrane. The FI subsequently refolds into the postfusion structure, driven by structural changes in RFH and chaperoned by GP, with RFH and HR2 providing leashes that pack the HR1-CH backbone grooves. (b) Model of the FI of the SARS-CoV-2 spike (S) protein, exact structure. Source structures for each S2 subunit domain are indicated, either SARS-CoV-2 prefusion (PDB: 6XR8), SARS-CoV-2 postfusion (PDB: 6XRA), or HR2 of SARS-CoV (PDB: 2FXP). Transmembrane domain (TMD) and cytoplasmic tail (CT) structures predicted by QUARK. (c) Comparison between predicted FI structure and known crystal structures of the prefusion (PDB: 6XR8) and postfusion (PDB: 6XRA) SARS-CoV-2 S2 subunit. One protomer highlighted in color. Dashed lines: missing domains from partially solved crystal structures. (d) Details of loaded spring transition. (e) β-hairpin (BH) domains in the known prefusion and predicted FI structures (boxed regions of (c)). The RFH domain is omitted from the FI BH for clarity. Following the loaded spring transition, HR1, CR, and FP (shown faint in the prefusion BH) vacate their prefusion locations in BH. The resultant cavity (arrow) would presumably be unstable. We assume the FI adopts the more compact postfusion BH structure (right). (f) The golden peptide (GP) domain chaperones refolding of the fusion intermediate (FI) into the postfusion structure. Blowups of boxed regions in (c) are shown. Refolding of the refolding hinge (RFH) domain is guided by GP. RFH forms a parallel β-strand with GP (red circle), the RFH unstructured portion packs the CH-GP groove, and RFH helices interact with two GP helices. Colored BH and CH belong to one protomer; colored RFH belongs to a different protomer.

Entry of SARS-CoV-2 is catalyzed by the trimeric S protein, each protomer having one S1 and one S2 subunit.^3^ The S protein has two cleavage sites. The S1/S2 cleavage site localized at the S1/S2 boundary (Figure 1a,b) is cleaved by furin in the virus-producer cells,^37,38^ so at this stage S1 and S2 are noncovalently associated. Following binding of S1 to the host cell ACE2 receptor,^39^ S1 dissociates from S2^40^ and the prefusion S2 trimer undergoes a major structural transition to its potent, fusogenic form, the fusion intermediate (FI)^5^ (Figure 1a).

We predicted the structure of the FI based on information from the partially solved pre- and postfusion cryo-EM structures^7^ (Figure 1b,c). A natural question is whether formation of the FI from its prefusion state uses a loaded spring mechanism similar to that used by influenza HA for the analogous transition.^36^ We hypothesized that in S2 a transition converts the prefusion heptad repeat 1 (HR1) domain into a continuous α-helix, itself continuous with the downstream central helix (CH). That is, all unstructured loop domains in HR1 become helical and rotate like torsional springs, straightening HR1 (Figure 1a,d). The result is a three-helix HR1-CH coiled coil in the FI trimer, the mechanical backbone of the FI. The hypothesis is tantamount to assuming CH and HR1 adopt their postfusion structures in the FI, since the postfusion HR1 and CH are continuous helices in a trimeric coiled coil^7^ (Figure 1c).

A second cleavage site is S2′, located within S2 (Figure 1a,b). S2′ is cleaved by the transmembrane serine protease TMPRSS2 at the host plasma membrane or by endosomal cathepsin L.^39,41^ The S2′ site is assumed cleaved before the loaded spring straightening transition, exposing the FP N-terminus ready for host membrane capture, consistent with SARS-CoV-2 lung cell entry being blocked^39^ by inhibition of TMPRSS2. This cleavage disconnects the domain upstream of the FP which we call the golden peptide (GP) domain (Figure 1a,b), but GP remains physically attached to S2.^7^ (We tested an uncleaved model with connected GP and FP, but the FPs were sequestered and unable to access the target membrane, Figure S1.)

We assumed the β-hairpin (BH) domain adopts its postfusion configuration in the FI. Straightening of HR1-CH pulls these domains away from their prefusion locations and would leave large destabilizing cavities in BH,^3^ favoring transition to the postfusion configuration where BH is raised to fill the cavities and assembles into a pyramidal base^7^ (Figure 1e). The same is assumed of GP, since a GP β-strand interacts strongly with an antiparallel β-sheet in BH^7^ (Figure 1a). GP also contributes two small helices, and a long helix in a CH coiled coil groove completing a six-helix bundle (Figure 1a,e) providing structural support at the base of the CH-HR1 backbone (see below).

Downstream of BH the refolding hinge (RFH) domain was taken as the prefusion structure, since in the prefusion structure both the RFH and HR2 domains are remote from the CH/HR1/CR/FP domains involved in the straightening transition that occurs when the prefusion structure transits to the FI (Figure 1a). It is thus unlikely that the RFH or HR2 domains in the FI are significantly different to their prefusion structures.^3^ We used the HR2 structure of SARS-CoV from NMR^42^ whose HR2 sequence is the same as that of SARS-CoV-2.^10^ The unknown transmembrane domain (TMD) and cytoplasmic tail (CT) structures were predicted by QUARK.^43^

These components were integrated into the predicted SARS-CoV-2 FI structure shown in Figure 1b (see Methods). The model implies that subsequent refolding of the FI to the postfusion conformation occurs by the unstructured N-terminal loop of the refolding hinge (RFH) domain folding into BH by contributing a β-strand to an antiparallel β-sheet (Figure 1a,f). The remainder of RFH folds back as a leash packing a GP-CH groove in the six-helix bundle, ending in a small helix that attaches between the two small GP helices of the other two protomers, oriented almost perpendicular to the HR1-CH backbone. Refolding is completed when the helical HR2 becomes partially unstructured to pack a second leash into a HR1-CH groove and supply one helix to a six-helix postfusion bundle with HR1, the fusion core.^7^

Note that the model of Figure 1b defines only the initial condition for our simulations, and some structures evolved considerably. We use the postfusion HR1-CH and BH structures, measured with a high resolution of 3.0 Å,^7^ and in simulations these structures changed very little (see below). By contrast, we find the FP-CR and RFH-HR2 domains (the “head” and “base” of the FI) are highly dynamic. For these, and for unstructured domains such as the flexible GP loops unsolved in the cryo-EM postfusion structure, the structures evolved significantly in simulations.

### All-Atom Simulation of the Fusion Intermediate

Using complementary atomistic and coarse-grained MD methods, we tested the FI model of Figure 1b and measured its configurational statistics and dynamics (see Methods).

**Figure 2:**
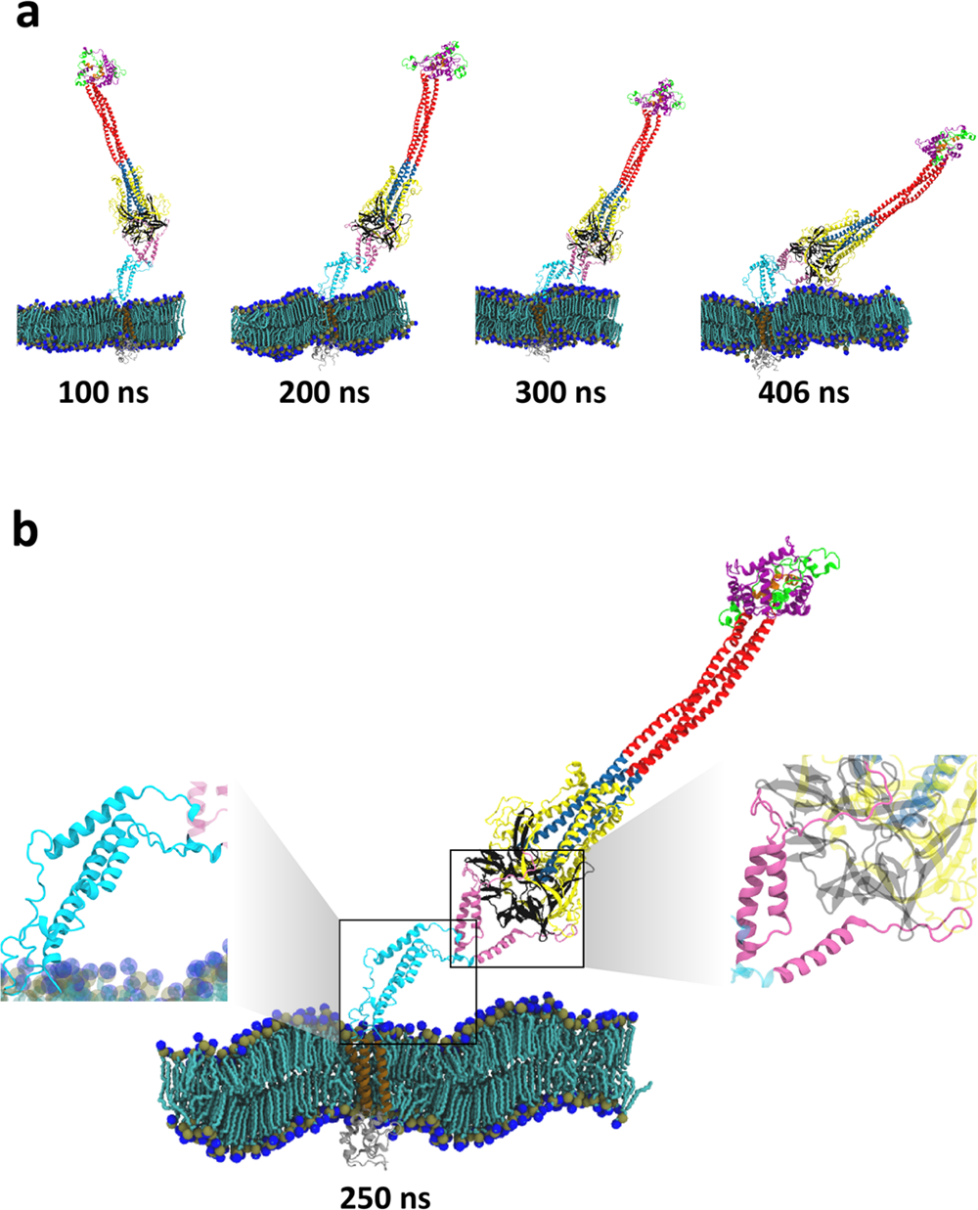
All-atom simulation of the SARS-CoV-2 fusion intermediate. Color code for this and all subsequent figures, as for Figure 1. In addition, the N-terminal helices of the fusion peptides are shown in orange. (a) Snapshots of the FI during the ∼0.4 μs all-atom simulation of the model of Figure 1b. The FI undergoes large bending and extensional fluctuations. (b) Snapshot of the FI after 250 ns of the all-atom simulation. The RFH and HR2 domains (highlighted) show secondary structural plasticity. Relative to the prefusion structure, the RFH stem helices splayed into an inverted tripod that behaves as a mechanical suspension system for the BH and GP domains and the HR1-CH backbone. The HR2 secondary structure is dynamic. Bending of the FI stretched the outermost HR2 helices, triggering partial conversion into unstructured sections.

During ∼0.4 μs of all-atom simulation the basic secondary structure remained unaltered (Figure S2), lending credibility to the model, while some tertiary structure was dynamic. Far from the rigid extended object one might anticipate given its long helical domains (Figure 1b), the FI was highly flexible and underwent large configurational fluctuations, adopting bent configurations without structural damage (Figure 2). The structural robustness was due to energy-absorbing features. The RFH-HR2 base region downstream of the BH domain was highly flexible, allowing large tilt (Figure 2b and Supplementary Movie 1). Relative to the prefusion structure the three RFH helices, known as the stem helices, became splayed with separated N-termini, in an inverted tripod suspension system that buffered large displacements of the upstream BH and backbone. The HR2 helices became partially unstructured, a structural plasticity that helped the FI tilt to greater angles at the membrane (Figure 2b).

### Three Hinges Endow the Fusion Intermediate with High Flexibility

Next we measured longer time FI dynamics using MARTINI coarse-grained simulations, which fix the secondary structure but access timescales 2 orders of magnitude beyond those accessible with all-atom simulations. We used MARTINI 2.2 force field^44^ with explicit solvent (see Methods for a brief discussion of MARTINI 2.2 and its appropriateness for the present simulations).

**Figure 3:**
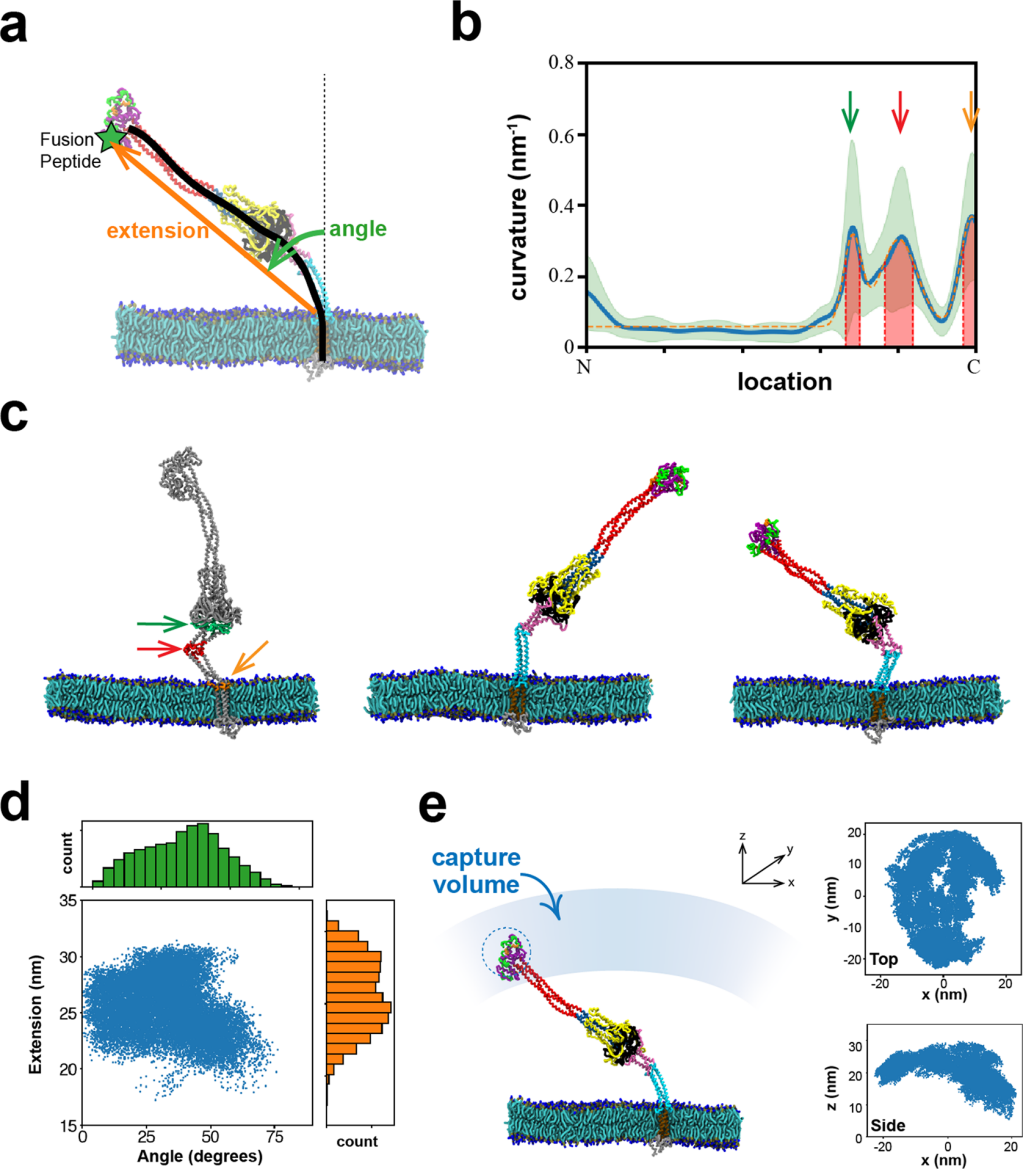
The fusion intermediate is highly flexible and visits a large capture volume. (a) In coarse-grained MARTINI simulations the FI had large configurational fluctuations, measured by the extension and angle of orientation of the FI backbone (black curve). (b) Time averaged backbone curvature versus normalized backbone arclength. The curvature is defined as the reciprocal of the radius of the osculating circle tangent to the fitted curve. Three high curvature hinges are apparent (arrows). Each hinge region (red) was defined as the quarter width of a fitted Gaussian (orange). Green envelope indicates standard deviation. (c) Simulation snapshots with the three hinges highlighted, identified as residues 1084–1138, 1156–1178, and 1204–1213. (d) Distributions of FI extensions and angles. (e) The FI has a large capture volume. Top and side views of FP locations visited. The FI extension and orientation ranges are ∼21–30 nm and of ±56°, respectively (95% of sampled values) so that a large capture volume is swept out over time, shown schematically (left). Dashed circle: approximate region explored by the FP in 1 μs. (b), (d), (e) Statistics are averages over the last 4 μs of five 8 μs runs, for a total of 20 μs simulation time.

In 40 μs total running time over 5 runs, the FI exhibited large configurational fluctuations as in the all-atom simulation, bending and reorienting over a wide range of angles (Figure 3a,c). To quantify the flexibility we measured the curvature statistics along the FI (Figures 3b, S3, Supplementary Movies 2,3, and Methods). This procedure identified three high flexibility hinge regions in the base, with similar mean magnitudes of curvature and large fluctuations (Figure S4). Mapping back to the atomistic structure located the hinges as unstructured loops at the BH/RFH, RFH/HR2, and HR2/TMD interfaces, respectively (Figure 3b,c).

These hinges have roughly the same locations as three hinges identified in the prefusion SARS-CoV-2 S protein by a study combining cryo-ET and molecular dynamics simulations.^27^ Thus, we adopt the “ankle, knee, hip” notation of that study. However, the hip hinge (RFH/BH interface) in the FI structure is more flexible than the prefusion hip due to the significantly altered RFH structure with splayed stem helices (Figure 2b).

To help assess whether these MARTINI-based long-time FI dynamics are consistent with all-atom dynamics, we compared the FI backbone stiffness and orientational fluctuations using both methods. The persistence length *L*_p_ of the HR1 part of the CH-HR1 backbone was *L*_p_ ∼ 62 nm and *L*_p_ ∼ 88 nm for MARTINI and all-atom, respectively (Figure S5). For equal sampling times (0.4 μs), backbone orientational fluctuations were greater in all-atom runs due to the more dynamical secondary structure in the base, but by 40 μs MARTINI simulations had sampled approximately the same angular range (Figure S6). Thus, atomistic and coarse-grained dynamics generated similar large base hinge bending motions, but all-atom simulations sampled the range of orientations more rapidly.

### Large Fluctuations of the Fusion Intermediate Lead to a Large Membrane Capture Volume

The first task of the unleashed FI is thought to be capture of the host target membrane by insertion of the fusion peptides at the protomer N-termini. Due to its flexibility the FI extension ranged from ∼21–30 nm (mean ∼26 nm), its orientation varied over angles ∼ ±60° to the membrane normal, and the FPs in consequence swept out a volume ∼25,000 nm^3^ at rate ∼750 nm^3^ μs^–1^ (Figure 3d,e, 95% of sampled locations).

Thus, due to the flexible base hinges combined with the large reach of the HR1-CH backbone, the FI accesses a substantial capture volume, equivalent to that of a ∼36-nm-diameter sphere. Following dissociation of S2 from the S1/ACE2 complex, this may help the virus rapidly reconnect with the host cell and limit refolding back into the virion membrane in a postfusion configuration without host cell contact. Indeed, postfusion spike proteins were observed by cryo-ET on intact SARS-CoV-2 virions.^47^

### Structure and Dynamics of the Membrane-Bound Fusion Peptide

**Figure 4:**
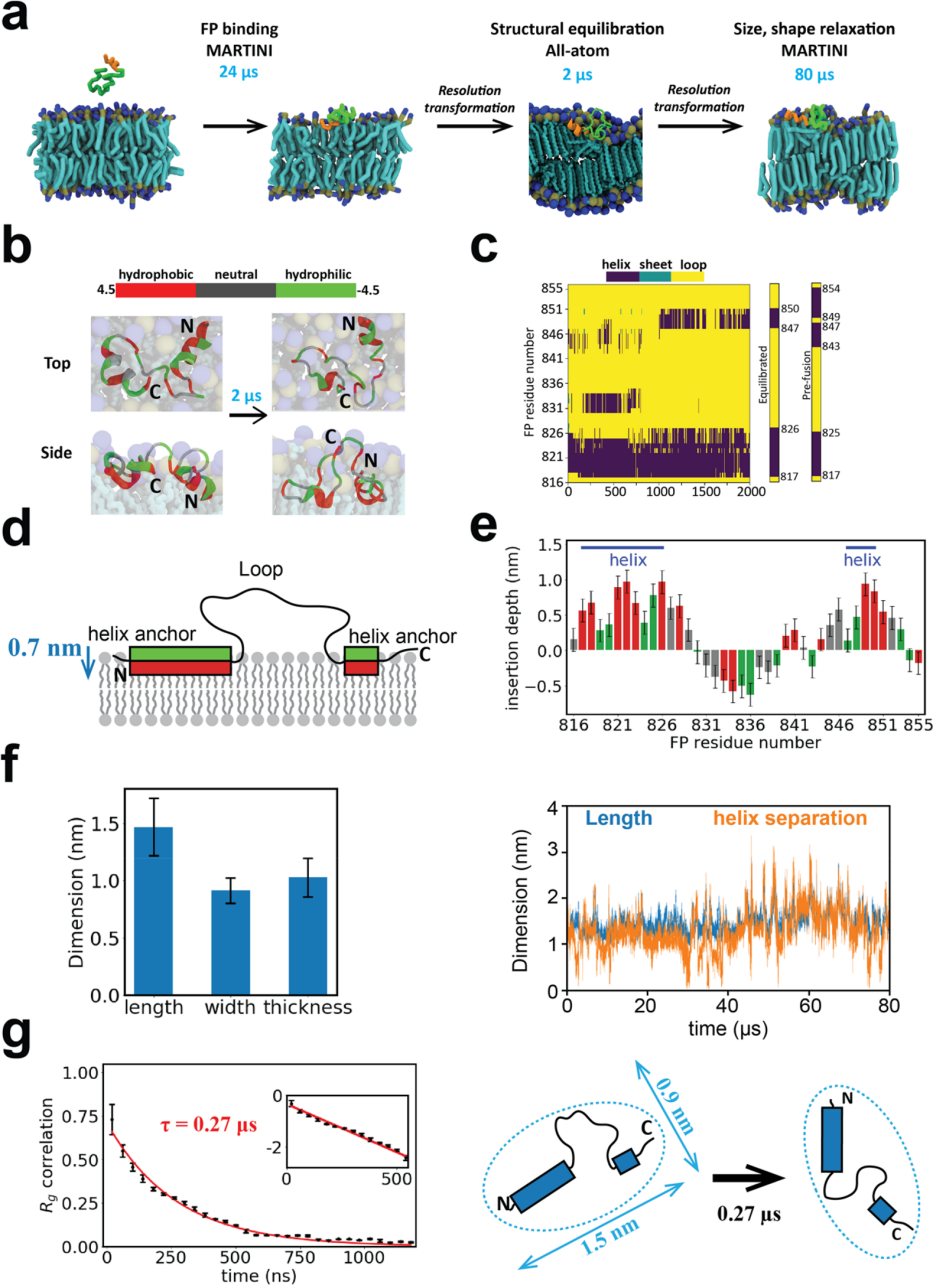
Multiscale simulations of the membrane-bound fusion peptide. (a) Multiscale simulation strategy to measure secondary structure and spatiotemporal statistics of the membrane-bound FP. The prefusion structure is coarse-grained to MARTINI representation and bound to and relaxed within the membrane in a 24 μs simulation (binding required ∼4 μs). Following backmapping to atomistic resolution, the secondary structure of the membrane-bound FP is relaxed in a 2 μs all-atom simulation. Assigning each residue its most frequently visited secondary structure during the final 0.8 μs of the all-atom simulation, the FP is again coarse-grained and its spatial dimensions and relaxation time measured in an 80 μs CG simulation. (b) Evolution of FP structure during the 2 μs all-atom simulation of (a). Initial and final states are shown. FP resides in one of three colors depending on the hydrophobicity. (c) Evolution of bound FP secondary structure during the 2 μs all-atom simulation of (a). The initial (prefusion) and final (equilibrated) structures are compared. For each residue the equilibrated structure shows the most frequently adopted in the final 0.8 μs. (d) Equilibrated bound FP following the all-atom equilibration of (a), schematic. The principal anchor is the amphiphilic N-terminal helix, with a secondary amphiphilic C-terminal helix anchor. Hydrophobicity color scheme as for (b). (e) Mean membrane insertion depth profile along the bound FP in the equilibrated structure represented in (c) (see Methods). Mean values over 0.8 μs. (f) Length and helix separation of the bound FP during the 80 μsMARTINI simulation of (a). Mean dimensions averaged over the final 78 μs (left). (g) Temporal correlation function of the radius of gyration of the bound FP yields shape memory time τ = 269 ± 1 ns. (Bin size, 40 ns. 100 data points per bin.) Inset: log-lin representation. Dashed lines: exponential fit. Top view, schematic (right). All error bars: standard deviations.

We used a multiscale approach to study the secondary structure and spatiotemporal statistics of the membrane-bound FP removed from its host FI (Figure 4a). The FI model of Figure 1b assumed the prefusion FP, but once bound its structure likely changes in the radically altered membrane environment. Thus, we used coarse-grained MARTINI molecular dynamics to bind and equilibrate the bound FP (24 μs total), followed by 2 μs of all-atom simulation to realistically evolve the bound secondary structure (Figure 4b).

During the all-atom simulation the secondary structure evolved (Figure 4b,c and Supplementary Movies 4,5). The N-terminus helix barely changed, but the two C-terminal helices merged into one. (The mean total helix content was 35%, compared to ∼20% from circular dichroism spectroscopy,^48^ a difference possibly explained by the differing simulated and experimental membrane compositions.) The equilibrated helices were amphiphilic and anchored the FP to the membrane with hydrophobic and hydrophilic residues oriented toward and away from the membrane, respectively (Figure 4d,e).

In an 80 μs coarse-grained simulation we then measured the statistics of the bound, equilibrated FP (Supplementary Movies 6,7). The depth of residues decreased somewhat (Figure S7a), and the C-terminal helix became repeatedly unanchored (Figure S8). The bound FP had root-mean-square (rms) length ∼1.5 nm and width ∼0.9 nm, defined as the greater and smaller of the gyration tensor eigenvalues in the *x*–*y* plane, while the rms thickness was ∼1.0 nm (Figures 4f, S9, and Methods). The FP extended with the anchored helices at either end, roughly speaking, as the length was strongly correlated with their separation. The radius of gyration autocorrelation function revealed a configurational memory time of ∼270 ns (Figure 4g), with similar times for the length, width, and thickness (Figure S10).

### Measurement of Fusion Peptide-Membrane Binding Rate Constant

**Figure 5:**
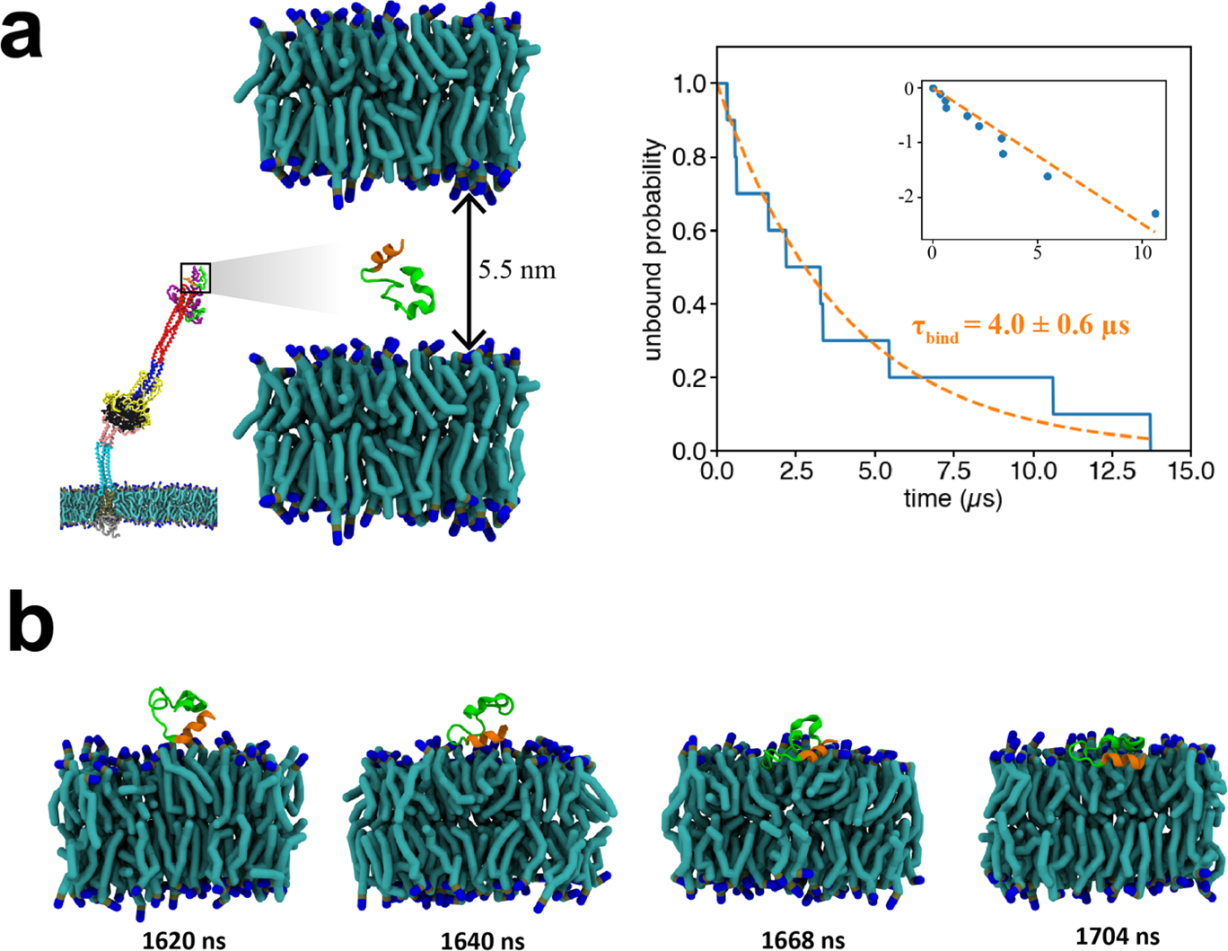
Membrane binding kinetics of an isolated fusion peptide. (a) Binding assay to measure the membrane binding rate constant, *k*_bind_^FP^, of a FP removed from its host FI. Initially the FP is positioned between two membranes separated by 5.5 nm (left). FP dynamics are simulated using the coarse-grained MARTINI force field, and the time to irreversibly bind the membrane is measured. The unbound fraction (blue, right) among ten simulated FPs decays exponentially with time constant τ_bind_ = 4.0 ± 0.6 μs (dashed orange curves). Inset: log-lin representation. (b) Typical binding event. The N-terminal helix (orange) is the first binding contact. To show secondary structure, the FP was back-mapped to all-atom representation.

The target membrane is captured by insertion of the fusion peptide. To quantify the binding kinetics, we removed a FP from its FI host and measured the binding rate constant between two membranes separated by *h* = 5.5 nm (Figure 5). The binding time itself is not an invariant quantity, as it depends on the proximity of the membranes.

We define a “collision” as a close approach of the FP to the membrane, such that the center of mass of the FP lies within the root-mean-square end-to-end distance *R*_FP_ ∼ 1.6 nm of the target membrane. The FP collided ∼27 times per μs with the membrane before irreversibly binding (Figure S11). Thus, effects of initial condition dependence and diffusion-control were negligible.^49^ Averaged over 10 coarse-grained MARTINI simulations the unbound probability decayed exponentially with time constant τ_bind_ ∼ 4.0 ± 0.6 μs (Figure 5a). The binding rate constant *k*_bind_ is defined by an imagined situation with a solution of FPs at density *c*_FP_ contacting a membrane, such that d*ρ*/d*t* = *k*_bind_*c*_FP_ where ρ is the areal number density of bound FPs.^49^ From the binding assay,
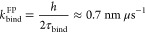
where the factor of 2 reflects the two membranes.

Importantly, binding was mediated by the N-terminal helix of the FP, which provided first contact with the membrane during a binding event (Figure 5b and Supplementary Movie 8). Since cleavage at the S2′ site would expose this helix, this is consistent with this cleavage being required for viral entry.^39^

Coarse-grained methods can probe much longer timescales, but dynamics may be altered. Since MARTINI dynamics are typically faster than all-atom dynamics due to the smoother coarse-grained energy landscape,^50,51^ for the binding constant above and elsewhere in this study we follow a standard procedure^52^ by reporting MARTINI simulation times as 4 times the raw value to account for the faster molecular diffusion (see Methods). In a recent all-atom simulation using periodic boundary conditions to effectively simulate membranes separated by ∼5 nm,^30^ SARS-CoV-2 FP binding times τ_bind_ < 300 ns were measured, with no binding after 300 ns in ∼30% of runs, suggesting an overall binding time τ_bind_ ∼ 0.5 μs. Given our ∼4 μs MARTINI binding times, this suggests the actual binding constant may be 5–10-fold smaller than the value above. Future systematic comparison of MARTINI and all-atom binding times under identical conditions would be of great interest.

### The Fusion Intermediate Captures Target Membrane on a Millisecond Timescale

**Figure 6:**
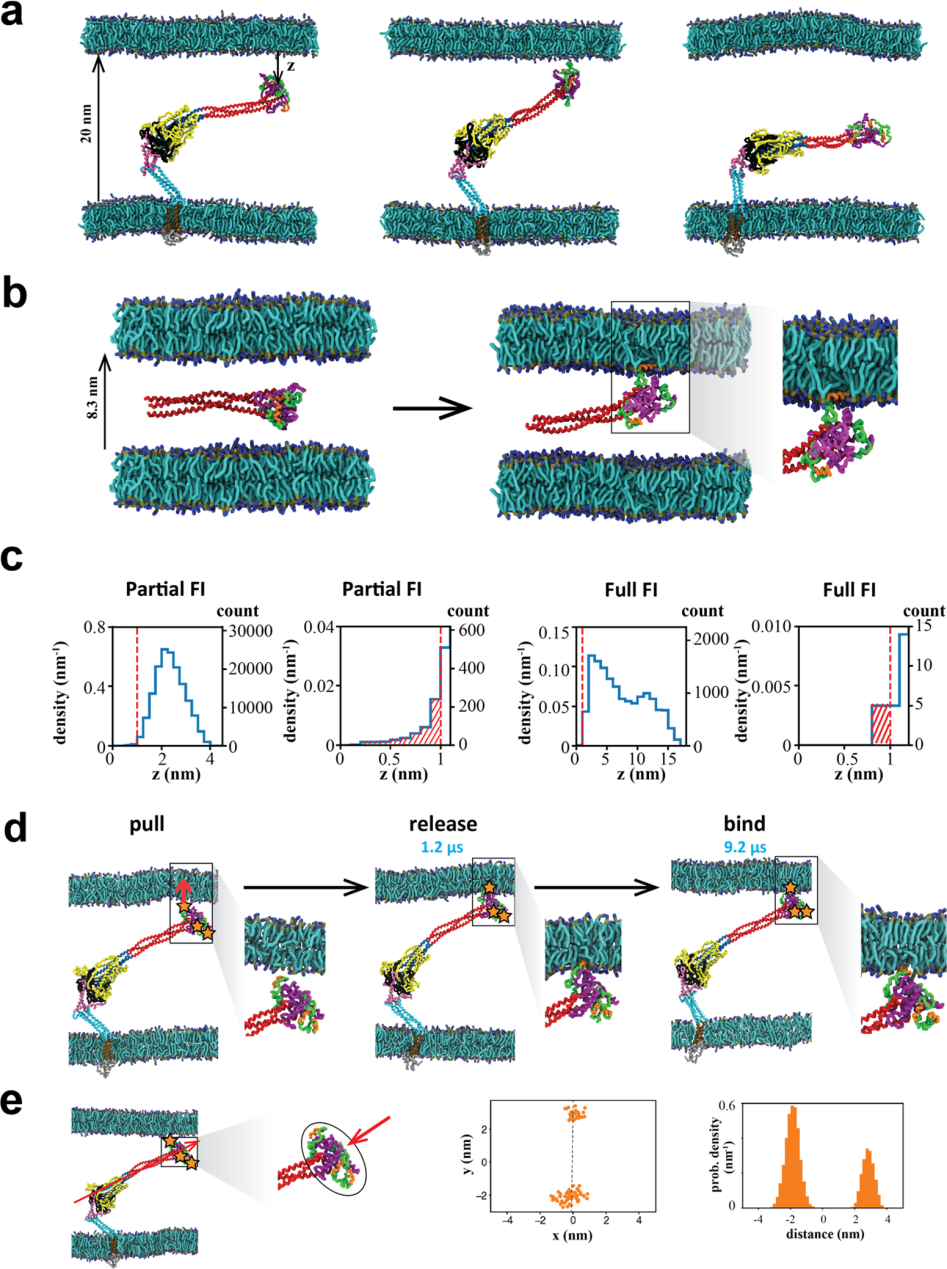
Interaction of the fusion intermediate with a target membrane. (a) Snapshots from coarse-grained MARTINI simulations of the full FI in the presence of a target membrane 20 nm from the viral membrane. The N-terminal helices of the FPs are shown orange. (b) Simulation of membrane binding by a truncated FI consisting of the HR1, CR, and FP domains between two target membranes. Initial condition (left). Binding was mediated by the N-terminal FP helix (right). (c) Probability density versus distance *z* of the nearest N-terminal FP helix from the membrane during simulations of membrane binding by the partial or full FI. For the partial FI, the plot at right shows a blow-up of the left plot for small *z* values. Similarly for the full FI plots. In both cases the density is depleted close to the membrane. The net probability for the N-terminal helix of the FP to lie within 1 nm of the membrane was 0.33% for the partial FI and 0.07% for the full FI (hatched areas). (d) Enforced binding of a FP. The FP N-terminal helix was pulled into the membrane over a period of 1.2 μs, and then released. The FP remained bound to the membrane for all of a 8 μs coarse-grained MD simulation. (e) The three FP-CR domains organize into a disordered laterally extended blob at the N-terminal end of the FI backbone, the FI head. Two FP N-terminal helices reside at one end of the head, one at the other end (orange stars). End view (perspective of red arrow) of N-terminal helix beads and their density distribution along the principal axis (dashed black line) in the plane normal to the backbone.

Next we studied membrane capture by the full FI (Figure 6a). Surprisingly, membrane binding was so much slower than suggested by the binding kinetics of the removed FP (Figure 5) as to be unobservable on available computational timescales. However, we observed binding of a truncated FI, from which we inferred a membrane capture time by the full FI of ∼2 ms.

We simulated the full FI in the presence of a target membrane 20 nm from the viral membrane (Supplementary Movie 9). Enabled by its high flexibility hinges, the FI adopted highly bent shapes in which the FPs were oriented toward the membrane (Figure 6a). However, we recorded no FP-mediated binding events during a total ∼300 μs of coarse-grained simulation over 20 independent runs (see Methods). Thus, binding is much slower when the FPs are attached to the FI. The FP-only binding kinetics are unrepresentative, as they suggest the FI will bind at rate ∼*k*_bind_^FP^*c*_FP_^1^(0) where *c*_FP_^1^(0) ∼ 1/Δ*z* is the 1D FP density evaluated at the membrane and Δ*z* ∼ 10 nm is the spread of FP N-terminal helix distances from the membrane (Figure S12). This yields a binding time τ_bind_ ∼ Δ*z*/*k*_bind_^FP^ ∼ 15 μs, clearly a huge underestimate.

It is unclear if binding is computationally accessible even with coarse-grained dynamics, given that longer than ∼0.3 ms is required. Thus, we accelerated the kinetics by truncating the FI, excluding all domains downstream of HR1. We measured the binding time of this partial FI, consisting of HR1, CR, and FP domains only, between two membranes separated by 8.3 nm (Figure 6b and Supplementary Movie 10). In two of six runs each lasting 160 μs, the membrane was captured by insertion of the N-terminal helix of one FP, after 23 and 133 μs. (In another case, binding was followed by unbinding after ∼42 μs.) This implies a best estimate of ∼390 ± 280 μs for the mean time for the partial FI to bind in this assay (see the Supporting Information, SI). Note that the orientation of the truncated FI backbone is somewhat more severely constrained in these runs (Figure 6b) than is the full FI backbone during an N-terminal helix-membrane collision (Figure 6a). However, since the full FI backbone is forced to orient nearly parallel to the membrane during a collision (the more so for membrane separations less than 20 nm), we believe that these truncated FI binding rates are qualitatively representative. A more complete analysis will be required to systematically average over membrane separations and backbone orientations.

To translate this result to binding of the full FI, we measured the fractions of time for which one of the three FP N-terminal helices lies within 1 nm of the membrane. The full FI satisfied this criterion ∼5-fold less frequently than did the partial FI (∼0.07% vs ∼0.33%, Figure 6c and Methods), suggesting membrane binding is ∼5-fold slower than in the partial FI assay. Thus, we estimate the target membrane is captured by the full FI after ∼2 ms.

Finally, to verify the full FI is capable of maintaining a bound state, we enforced binding by pulling the FP of an FI into the membrane (Figure 6d). The FP remained stably bound for all of an 8 μs simulation.

### Fusion Peptides and Connecting Regions Form a Disordered Cluster

These results show that the ability of the FP to access target membrane is strongly constrained by its local environment in the FI. In the coarse-grained simulations this environment was a disordered cluster that the FPs and neighboring CR domains organized into, laterally extended at the N-terminal end of the HR1-CH backbone (Figure 6e). We call this the head of the FI. Two of the three FP N-terminal helices resided at one end of the head, and one more exposed helix at the other. With the host membrane ∼20 nm away, likely imposed by the earlier S1-ACE2 binding episode, the FI is severely bent (Figure 6e). The lateral orientation of the head appears optimal for presenting the helices to the membrane for binding in this bent configuration.

### Simulations Reproduce Massive Fluctuations of Fusion Intermediates Observed in Cryo-ET

**Figure 7:**
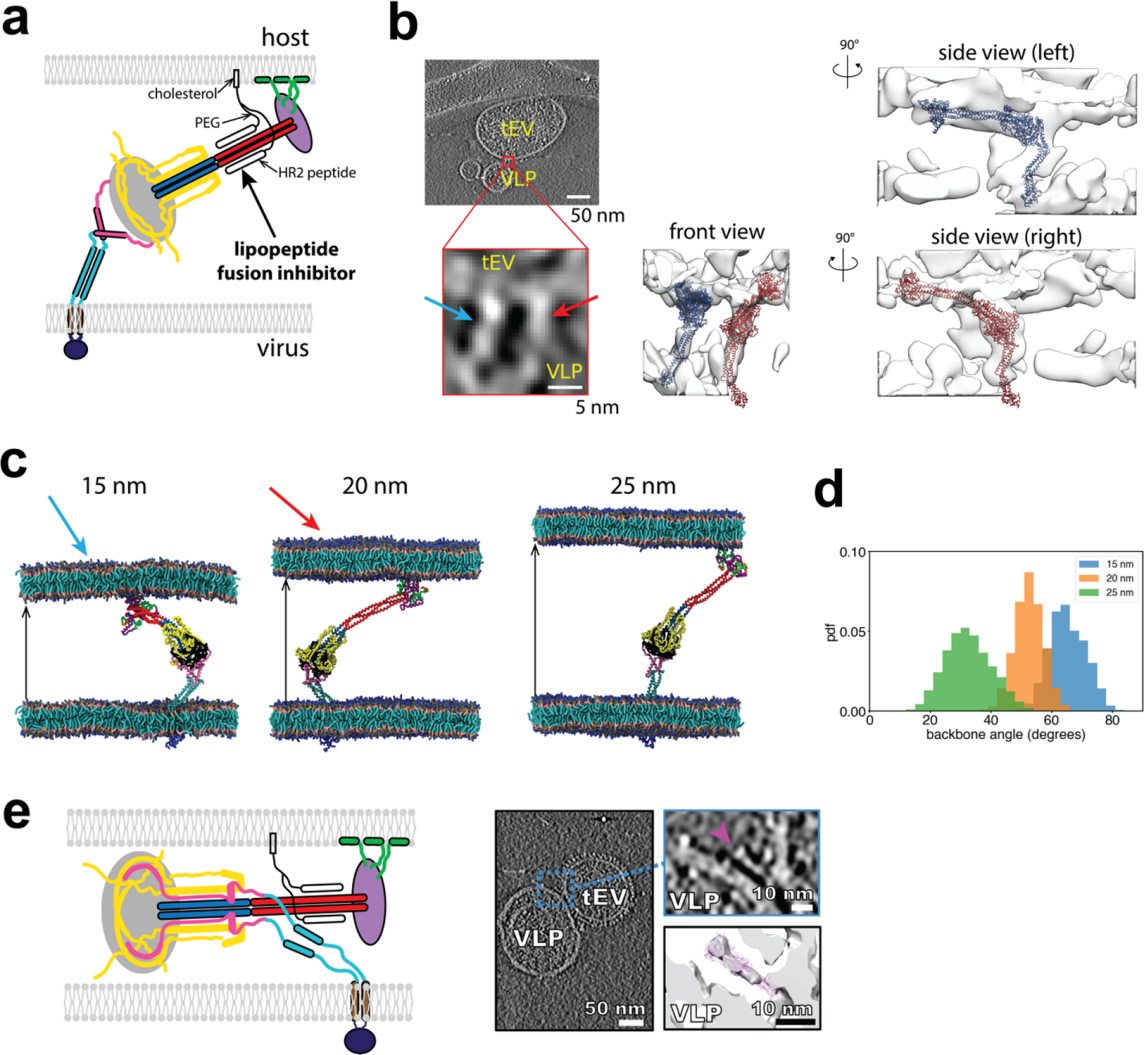
Comparison of simulated fusion intermediate configurations to cryo-ET density maps. (a) Scheme used in the cryo-ET experiments of ref (53). The FI is stabilized by a lipopeptide^19^ anchored to the target membrane via a cholesterol group. The HR2-derived peptides of the inhibitor bind HR1 of the FI (red), blocking HR2-HR1 binding and the final step of FI refolding. (b) Cryo-ET images from ref (53), showing FIs that span the membranes of virus-like particles and target extracellular vesicles. A tomogram z-slice (left) shows two flexible FIs connecting the membranes (arrows). The corresponding 3D density map was used to find the best fit structure from simulations; front and side view perspectives of the FI at left and at right, respectively, are shown in the isosurface representation, middle and right. The side views reveal the long FI backbone. The cryo-ET images were reproduced with permission from ref (53). Copyright 2022, Science Advances. (c) Snapshots from CG MARTINI simulations of membrane-spanning FIs for three separations. The structures used to fit the cryo-ET densities of (b) are indicated (arrows). (d) Distributions of FI backbone angles relative to the target membrane for three membrane separations. (e) Schematic of the partially refolded intermediate in the presence of the lipopeptide (left). Cryo-ET images^53^ of a partially refolded intermediate (right). Virus-like particles (VLP) and a target extracellular vesicle (tEV) are indicated. The cryo-ET images were reproduced with permission from ref (53). Copyright 2022, Science Advances.

To directly test our model experimentally, we compared simulated FI configurations with configurations measured by cryo-electron tomography (cryo-ET). Our simulations predict the FI captures the target membrane within milliseconds, suggesting that experimental visualization of the unbound extended FI during this macroscopically small time window is difficult. Thus, we compared instead to experimental measurements of target membrane-bound FIs. We recently used spike-containing virus-like particles interacting with ACE2-containing target extracellular vesicles to capture the spike FI. To arrest the FI, we used a previously developed lipopeptide fusion inhibitor that blocks the refolding of the FIs. This enabled us to use cryo-ET to visualize the extended SARS-CoV-2 FI for the first time^53^ (Figure 7a). The measured cryo-ET densities suggested large configurational fluctuations of the FIs (Figure 7b).

To model this experimental situation, we performed new coarse-grained MARTINI simulations of the full-length FI, with its TMD bound to the viral membrane and one FP bound to a second target membrane a distance 15, 20, or 25 nm away from the viral envelope (Figure 7c), consistent with the separations in the cryo-ET experiments.^53^ Binding was initiated by pulling the FP into the target membrane using the procedure previously described (Figure 6d). The membrane-bound FI was then simulated for 20 μs to generate FI configurations for comparison with cryo-ET densities. Simulated FIs executed large orientational fluctuations but always remained stably bound (Figure 7d).

From the 3D cryo-ET density maps for the data of ref (53) we identified nine membrane-spanning FIs from seven tomograms. For each of these configurations we sought a best-matched simulated FI structure sampled from three 20 μs runs, by first selecting simulated FIs whose end-to-end distances (between the TMD and FP anchoring points) matched the experimental value, and then rotating for best fits (Figure 7b). The simulated FI maximizing the Pearson correlation between the simulated and cryo-ET density distributions was deemed the best overall fit (see Methods). Seven of the nine experimental FIs closely matched simulated FI configurations (Pearson correlation >0.15) and included a randomly oriented elongated tubular section (Figure 7a) corresponding to the HR1-CH backbone. The remaining two experimental structures were apparently branched, which we interpret as the superposition of a pair of FIs.

Overall, the comparison quantitatively supports the basic model of Figure 1 and the FI structures emerging from simulations, including massive hinge-mediated fluctuations.

## Conclusions

The outbreak of COVID-19 saw rapid efforts to characterize the pre- and postfusion SARS-CoV-2 spike protein structures,^3,7,9,10^ but the structure of the fusion intermediate (FI) that facilitates fusion and entry remains unknown and the pathway to fusion and entry is poorly characterized. The first step on this pathway is capture of the host cell target membrane by the FI, but the mechanism and timescale are unknown for SARS-CoV-2 or indeed any coronavirus.

Here we built a full-length model of the SARS-CoV-2 FI, extrapolating from pre- and postfusion structures^7^ (Figure 1a). From coarse-grained simulations we inferred a FI-mediated membrane capture time of a few milliseconds. Macroscopically this is fast, suggesting that therapeutic strategies targeting the FP are limited by a small ∼millisecond window during which the FP is exposed, and that targeting the refolding process^10,19,54^ may be more fruitful. Indeed, refolding may be a relatively slow step, as suggested by our previous observation of unfolded target membrane-bound extended FIs in cryo-ET images,^53^ despite the fact that the lipopeptides in this study presumably permit refolding other than the final refolding episode in which HR1 and HR2 form a six-helix bundle. Consistent with this view, some of the experimentally observed structures appear to be in advanced stages of refolding^53^ with structures presumably close to the postfusion S2 structure, except the membranes are not yet fused, so the final stages of refolding have not yet occurred (Figure 7e). Future measurements of unfolded target membrane-bound SARS-CoV-2 FI lifetimes would be most interesting. For influenza hemagglutinin, it was suggested the extended FI may have a ∼1 min lifetime limited by the waiting time for activation of additional FIs for cooperative refolding and membrane fusion.^11^

While millisecond membrane capture is macroscopically very fast, from a computational perspective the timescale is very long: given our computational resources, membrane capture would require several hundred years of atomistic simulation (see Methods), and so is observable only with coarse-grained molecular dynamics methods. Membrane binding rates were overestimated ∼2 orders of magnitude by simulations with the FP removed from its host fusion protein, although a 10 residue N-terminal helix directing and maintaining FP binding was identified (Figure 5b) in accord with a recent study.^29^ Simulations of isolated viral fusion peptides are commonly implemented,^29−31,55^ but our results suggest they should be interpreted with caution as the fusion peptide environment is radically altered when attached to its host fusion protein.

We predicted here that the FI captures the host cell membrane after ∼2 ms. However, given the giant fluctuations of the highly flexible FI seen in cryo-ET experiments^53^ and our simulations, we expect large variations about this mean value. Furthermore, since MARTINI by its nature fixes secondary structure, the MARTINI simulations used to simulate membrane capture fail to fully capture the dynamic nature of the hinges (the RFH and HR2 domains). MARTINI does indeed capture the splaying of the RFH helices, but not the local unfolding of HR2 seen in all-atom simulations (compare Figure 6 to Figures 2, S2). Thus, the FI may in reality have even more dynamic hinges than captured by MARTINI, suggesting a somewhat shorter capture time than 2 ms.

**Figure 8:**
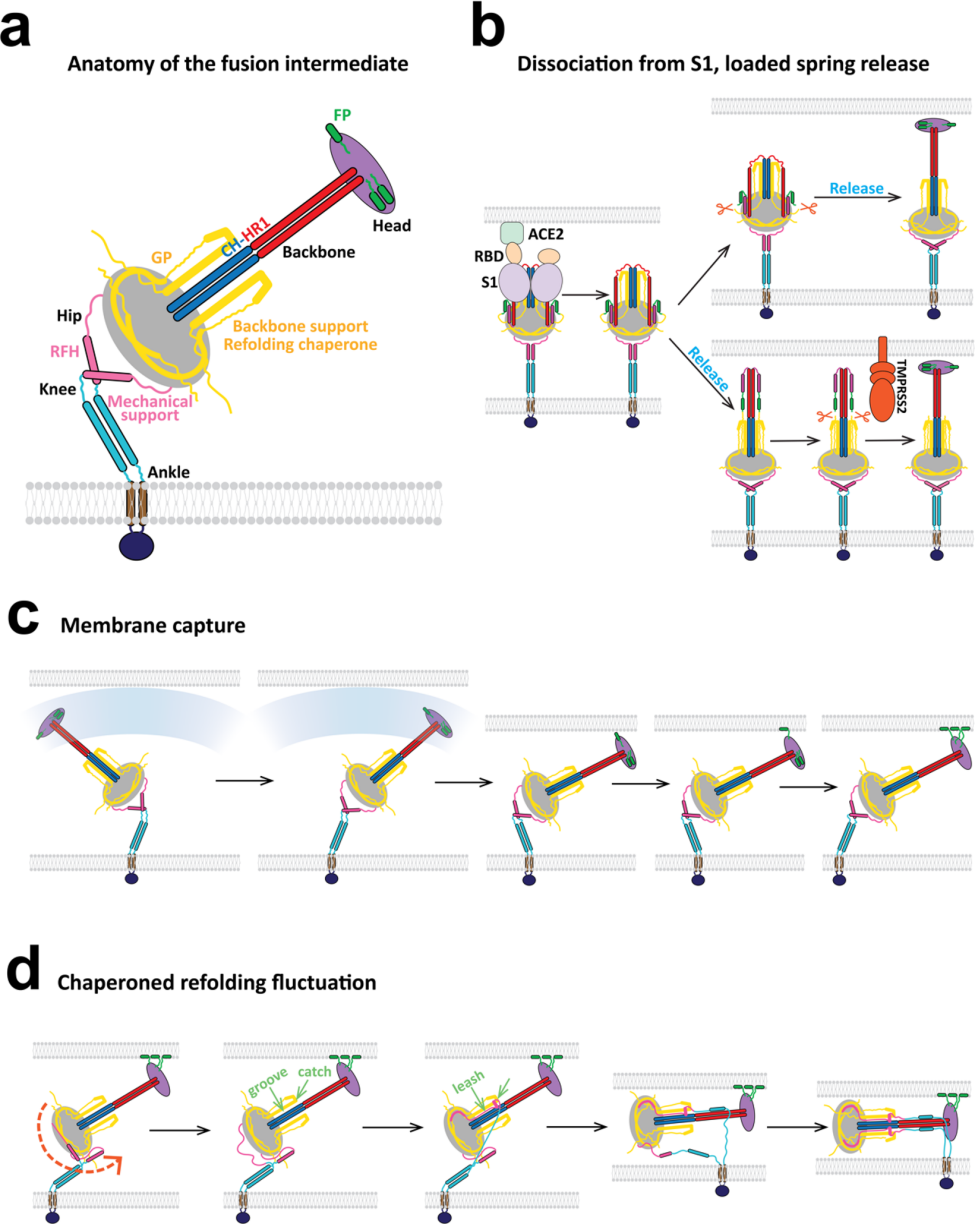
Model of the SARS-CoV-2 fusion intermediate and the pathway to fusion. (a) Schematic of the fusion intermediate. The ankle, knee, and hip hinges impart high flexibility to the FI. RFH is an inverted tripod suspension system buffering longitudinal backbone fluctuations. GP supports the backbone and chaperones refolding. The CH-HR1 backbone provides mechanical strength and reach. The FP-CR head houses the fusion peptides for host membrane capture. (b) Pathway to the fusion intermediate. Following dissociation of S2 from the S1/ACE2 complex, a loaded spring release mechanism generates the fusion intermediate after proteolytic cleavage at the S2′ site (upper pathway) or before cleavage (lower pathway). RBD, receptor binding domain of S1. TMPRSS2, transmembrane protease serine 2. (c) Schematic of host cell membrane capture by the fusion intermediate. Three base hinges endow the fusion intermediate with high flexibility and large configurational fluctuations, so the N-terminal fusion peptides sweep out a large volume for membrane capture. (d) Model of fluctuation-triggered, GP-chaperoned refolding. A sufficiently large rotational fluctuation at the RFH/BH hip joint unfolds a RFH stem helix into an unstructured loop. The loop is grabbed by a GP β-strand in BH, initiating RFH refolding, and guided into a GP-CH groove which it packs as a leash. Leash zippering into the groove is stabilized by the GP catch, preventing unzippering. Refolding of the HR2 leash completes refolding of one protomer, pulling the membranes together and helping the other protomers refold. The trans postfusion structure catalyzes membrane fusion in cooperation with other refolded fusion proteins.

From atomistic and coarse-grained simulations, the extended FI emerges as machinery with a specific design (Figure 8a) that executes efficient membrane capture (Figure 8b,c) and pulls the membranes together (Figure 8d) ready for the final fusion step. The extended FI is a highly dynamic state with no single configuration, in contrast to the pre- and postfusion SARS-CoV-2 structures. Rather, the FI constantly undergoes huge configurational fluctuations, which we showed are consistent with cryo-ET measurements^53^ (Figure 7b). These large bending and tilting fluctuations are primarily due to 3 highly flexible hinge regions (Figures 2, 3, 6), similar to 3 hinges identified in the prefusion structure that were proposed to aid receptor binding.^27^ We suggest the hinges are most critical to the FI. Large fluctuations may aid capture of host cell membrane by enlarging the region accessible to the FPs at the FI terminal (Figure 8c) and may help to coordinate capture by multiple FIs at different distances. Indeed, influenza, para-influenza, and HIV-1 appear to use several FIs.^12,14,56^ Further, by allowing the ∼25-nm-long FI to bend significantly, the extreme flexibility may facilitate the prefusion-to-FI transition even in the confined circumstances of a nearby host cell membrane (Figure 6a) and allow the FI to tilt its head and present the FPs directly to the target membrane (Figures 6e and 8c). A milder flexibility was observed in the prefusion HA of influenza, which was reported to bend through ∼25° mediated by a linker between the ectodomain and TMD.^57^

Following binding of the FI to the target membrane on a millisecond timescale, the next step on the pathway to fusion and cell entry is refolding of the FI that pulls the viral and target membranes together. What sets the timing of refolding? Refolding requires a major structural transition of the RFH domain between the hip and knee joints just upstream of the β-hairpin (BH) domain (Figure 1a, 8a). The RFH domain must fold into the GP domain and the CH-HR1 backbone (Figure 8d). In all-atom and coarse-grained simulations the RFH domains had a highly dynamic structure, permitting large bending fluctuations of the hip hinge at the RFH/BH interface (Figures 2 and 6a). This suggests the refolding time may be the waiting time for a rotational hip hinge fluctuation sufficiently large to destabilize one of the splayed RFH helices into an unstructured loop (Figure 8d). The loop would be highly susceptible to GP-chaperoned refolding. FI refolding and the drawing together of the host and viral membranes may be mutually reinforcing elements in a cooperative process, as a smaller membrane separation presumably favors refolding, while refolding decreases the membrane separation.

As the machinery that achieves cell entry, the FI is a natural therapeutic target. A number of candidate drugs have targeted the refolding step. HR2-derived peptides inhibit fusion by SARS-CoV-2 and MERS-CoV, presumably blocking formation of the HR1-HR2 six-helix fusion core.^10,19,58^ Their efficiency as fusion inhibitors is insensitive to mutations in the spike protein,^23^ suggesting potential as robust antiviral drugs.

Understanding the mechanisms of such candidate drugs and discovery of new FI-targeting drugs will be helped by establishing the structure and dynamics of this elusive intermediate. For example, another potential target is the golden peptide (GP) domain extending from the S1/S2 to the S2′ cleavage site (Figure 1). In addition to its structural role as a stabilizing socket for the CH-HR1 backbone (Figure 8a), GP chaperones RFH refolding (Figure 8d). First, GP helps initiate RFH refolding by providing the β-strand that the RFH N-terminus loop folds onto as a parallel β-strand. Second, GP supplies a groove with the neighboring CH helix into which the RFH leash packs, continuing refolding. Third, a small RFH helix is pinned by the GP catch, a U-shaped sequence including 2 small helices, that may rectify zippering of the RFH leash by preventing its unraveling from the groove. Thus, GP- or RFH-derived peptides could inhibit FI refolding by binding the RFH or GP domains. Such peptides might also stabilize the short-lived unfolded FI for visualization.

Interestingly, a recent study identified an 8-residue region in the prefusion RFH stem helix as the epitope of two cross-reactive monoclonal antibodies.^59^ This region becomes the small RFH helix that engages the GP catch during refolding, together with the four downstream residues. Thus, the antibodies may neutralize SARS-CoV-2 by binding RFH and interfering with the GP catch that rectifies RFH refolding. Our simulations suggest another possibility is that binding of the RFH stem helices in the FI alters their dynamics and lowers the hip hinge flexibility, with possible consequences for membrane capture and/or refolding. This would be similar to the effect of antibodies targeting the linker domain adjacent to the TMD of HA, which reduced the linker flexibility and suppressed orientational fluctuations.^57^

In summary, the extended FI is the fusogenic form of the spike protein that captures host cell membrane for fusion and entry and is a critical but relatively unexplored therapeutic target. Our model suggests a loaded-spring mechanism generates the FI from the prefusion structure, related to the mechanism for HA of influenza.^13,36^ The FI has unexpectedly large bending fluctuations that help it capture membrane in a few milliseconds and may trigger the refolding transition that draws the viral envelope and host membranes together for subsequent fusion (Figure 8). These results provide an account of a critical episode during cell entry and offer a framework for rational design of new therapeutic strategies to disable the FI.

## Methods

### Building a Complete Structure for the Fusion Intermediate of the SARS-CoV-2 Spike Protein

The primary sequence of the SARS-CoV-2 S protein was obtained from the NCBI database (GenBank: MN908947). We first built the HR1-RFH portion of the FI (including the associated GP domain). We used MODELLER^60^ with two specified templates: the postfusion structure (PDB: 6XRA) for HR1-BH and GP, and the prefusion structure (PDB: 6XR8) for RFH. Several constraints including 3-fold symmetry and preservation of secondary structure were specified. The C-terminal domains (HR2, TMD, and CT) were then appended to the HR1-RFH portion one by one using MODELLER. The source for HR2 was a SARS-CoV HR2 NMR structure (PDB: 2FXP), while the TMD/CT structure was predicted by the QUARK server by providing the primary sequences. The FP and CR were then extracted as a whole from the prefusion structure (PDB: 6XR8) and appended to the HR1 domain in the FI with an arbitrary angle using Pymol. Finally, a complete GP structure was made in the FI using MODELLER, by appending the N-terminal portion (residues 686–702) and C-terminal portion (residues 771–815) of the prefusion structure to the solved postfusion GP (residues 703–770).

### Multiscale Molecular Dynamics Simulations

To simulate the FI and its FP we used two complementary molecular dynamics approaches, all-atom and coarse-grained MARTINI. For the coarse-grained simulations we used the Martini 2.2 model. Other MARTINI-based models such as ELNEDIN and GoMARTINI implement additional local potentials (∼1 nm for ELNEDIN and the van der Waals radius for GoMARTINI) among backbone beads to constrain their relative locations and stabilize protein tertiary structure.^45,46^ In general the classical MARTINI 2.2 force field we use can lead to unphysical instabilities in tertiary structure. However, the structure of the FI is highly dynamic. For example, in MARTINI simulations of the membrane-bound FP, for the backbone beads of the FP relative to an initial condition we measured a root-mean-square deviation (RMSD) of ∼0.7 nm (Figure S13), greater than a typical ∼0.3 nm RMSD value for ELNEDIN and GoMARTINI. This is as expected, reflecting the fact that the bound FP has no fixed tertiary structure. More significantly, the FI has giant global fluctuations on ∼10 nm scales with no stable folded structure. Thus, the classical MARTINI 2.2 force field is expected to capture the essential features of the FI and its global dynamics.

Pure DPPC membranes were used to represent the viral envelope and the host cell membrane for simplicity. All simulation systems were solvated with 150 mM NaCl at neutral pH. Each simulation system was energy-minimized and equilibrated before the production run, which was performed in the NPT ensemble at 1 bar and 310 K using GROMACS 2019.6.^61,62^ The all-atom simulation of the FI (Figure 2) required 1 day of computation to run the FI for 10 ns. At this rate, ∼500 years would be needed to achieve the ∼2 ms timescale of membrane capture (Figure 6c). For details of the simulations and the analysis see SI.

### All-Atom Simulations

For the FI simulation (Figure 2), the FI model structure (Figure 1b) was placed in a planar lipid bilayer in a 16 × 16 × 43 nm^3^ box using the CHARMM-GUI membrane builder.^63^ The production simulation was run for 406 ns using the CHARMM36 force field.^64,65^ Secondary structure was identified using the *dssp* algorithm.^45,66^

To simulate the membrane-bound FP (Figure 4b), the final configuration of the FP and the membrane to which it was bound in a given CG FP binding simulation was converted to atomic resolution in CHARMM36 force field^64,65^ using the *backward* utility.^67^ The production simulation was run for 2 μs. The membrane insertion depth of each FP residue was defined as the vertical distance between the center of mass of the residue and that of the PO_4_ groups in the membrane leaflet to which the FP was bound.

### Coarse-Grained Simulations

All running times reported here are 4 times the raw MARTINI running time, to compensate for the faster sampling of the MARTINI model.^52^ Time-dependent data in MARTINI simulations were analyzed after adjusting in this fashion.

For the FI simulations in the absence of a target membrane (Figure 3), the FI model structure (Figure 1b) was first mapped into the MARTINI coarse-grained representation using the *martinize* utility^44,68^ and then placed in a planar membrane in a 30 × 30 × 50 nm^3^ box using the *insane* utility.^69^ Five production simulations starting from the same equilibrated system were run for 8 μs.

For the isolated FP-membrane binding assay (Figure 5), the atomistic structure of the FP was extracted from the prefusion cryo-EM structure (PDB: 6XR8) and coarse-grained into MARTINI representation. Then the FP was placed ∼1 nm above a planar bilayer in a 7 × 7 × 10 nm^3^ box. By implementing periodic boundary condition, this is equivalent to using two planar membranes separated by ∼5.5 nm. The C-terminal carboxyl group was neutralized as it connects to the CR in the full-length FI. Ten 24 μs parallel runs were performed. Binding events were identified by comparing the centers of mass of the FP and the PO4 beads in each membrane leaflet (see SI).

To simulate an equilibrated FP bound to a membrane (Figure 4), the final configuration of the FP and the membrane to which it was bound in the all-atom simulation was coarse-grained into MARTINI representation. The production simulation was run for 80 μs. In a variation of this procedure, the FP C-terminal helix was transformed to an unstructured loop by changing the secondary structure file of the FP, an input to the coarse-graining utility. We then measured the membrane insertion depth of each residue, gyration tensor, radius of gyration, length, and width of the FP (see SI).

For ten of the simulations of the FI interacting with a target membrane, ten snapshots from three FI simulations without membranes were used as initial conditions, in which the FI ectodomain protruded less than 20 nm normal to the membrane. Another pre-equilibrated planar membrane (run for 4 μs) was placed 20 nm above the membrane anchoring the FI, and this configuration was run for 8 μs. These runs were used to calculate the probability distributions of Figure 6c. Ten additional simulations (each lasting 22.4 μs) used a biased initial condition with the center of mass of the nearest N-terminal FP helix within 1 nm of the membrane and the unpaired helix facing the membrane. All simulations mentioned in this and the following paragraph were run in a box of 30 × 30 × 50 nm^3^. Here, the membrane position was defined as the mean location of all PO_4_ beads in the lower leaflet of the upper membrane.

To pull a FI-attached FP into a target membrane (Figure 6d), an initial condition was chosen with the nearest unpaired N-terminal FP helix lying within 1 nm of the membrane. The helix was then pulled toward the target membrane at speed 2.5 nm/μs for ∼1.2 μs, until the centers of mass of the helix and membrane were separated by ∼0.1 nm in the *z* direction. The pulling force was then released, and the simulation was run for 8 or 20 μs.

For simulations of membrane binding by the partial FI (Figure 6b), the structure of the HR1, CR, and FP domains in the MARTINI coarse-grained representation was extracted from the final configuration of a FI simulation. This partial FI was positioned above a planar bilayer in a 20 × 20 × 13 nm^3^ box with periodic boundary conditions, equivalent to two planar membranes separated by ∼8.3 nm. The C-terminal carboxyl group was neutralized. Six 160 μs parallel runs were performed. Binding events were identified similarly to the isolated FP assay (see SI). To infer the binding time of the full FI from the measured partial FI binding time, we assumed the binding probability per unit time for a given FP-CR-HR1 configuration is independent of the remainder of the FI.

### Fitting Simulated FI Configurations to Cryo-ET Density Maps

Tomograms of virus-like particles and target extracellular vesicles were obtained as previously described.^53^ In ImageJ, the data was further Gaussian filtered with a radius of 2*d* where *d* = 0.6484 nm is the cryo-ET voxel size, and then the image contrast was enhanced by 0.3%. Isosurface representations of the cryo-ET data were visualized in Chimera, from which we found nine potential FIs connecting the membranes of virus-like particles and extracellular vesicles. For each of these FIs, the end-to-end distance between the two membrane-anchor points was calculated. For the three 20-μs coarse-grained MARTINI simulations, the FI was equilibrated for 1 μs and the FI configurations were extracted every 10 ns from subsequent trajectories of 19 μs. Among these FI configurations a subset with end-to-end distance close to the experimental values was selected for fitting to the cryo-ET density. For each of these simulated FI candidate configurations, a simulated cryo-ET density was generated, by summing Gaussian point spread functions centered on the backbone bead locations of all residues, with widths *d,d*,3*d* in the *x,y,z* directions, respectively, of the experimental cryo-ET density map. The large width in the *z* direction accounts for missing wedge effects. Then the positions and rotation angles of the FI simulation candidates were optimized to maximize the Pearson correlation between the simulated and experimental density map. Optimization was repeated for each simulated FI candidate, and the candidate with the highest Pearson correlation was deemed the best fit structure.

## Data Availability

Simulation results supporting the findings of this paper, and codes to perform the simulations, to analyze the data, and to generate the technical figures are available in the Zenodo repository (https://zenodo.org/record/7487128#.Y6snOS2B2MI). The codes are also available in the GitHub repository (https://github.com/OShaughnessyGroup-Columbia-University/sars-cov-2-fusion-intermediate).

## Supporting Information Available

Supplementary methods (S2-S6), figures (S7-S19), and references (S21) (PDF)
Supplementary movies: Movie 1. All-atom simulation of the SARS-CoV-2 fusion intermediate. The color code is as for Figure 2. Movie 2. Coarse-grained MARTINI simulation of the SARS-CoV-2 fusion intermediate. The three hinges are colored according to the code of Figure 3c. Movie 3. Simulation of Movie 2 with the fitted curve. A fitted curve (red) to the ectodomain backbone of the fusion intermediate (blue) whose TMD is anchored in the viral envelope (green). Movie 4. All-atom simulation of a membrane-bound fusion peptide (side view). The residues are colored according to their hydrophobicity, using the color scheme of Figure 4b. Movie 5. Simulation of Movie 4, top view. Movie 6. Coarse-grained MARTINI simulation of an equilibrated fusion peptide bound to a membrane (side view). The N-terminal helix (orange) was always buried in the membrane, but after ∼52 μs of the simulation the C-terminal helix (purple) transiently unanchored from the membrane for ∼0.3 μs. Movie 7. Simulation of Movie 6, top view. Movie 8. Binding assay for an isolated fusion peptide (coarse-grained MARTINI simulation). The fusion peptide, removed from the FI, becomes bound to the membrane after ∼1.6 μs, with the N-terminal helix (orange) providing first stable contact. The fusion peptide remains bound for the remaining ∼22.4 μs of the simulation. The binding events of Figure 5b are snapshots from this movie. Movie 9. Simulation of the fusion intermediate interacting with a target membrane, 8 μs duration. One of the fusion peptide N-terminal helices (green spheres) repeatedly approaches the membrane to within 1 nm, but fails to bind. Figure 6e shows a snapshot from this movie. Movie 10. Binding assay of a partial fusion intermediate (coarse-grained MARTINI simulation, first 50 μs). The partial FI consisting of the FP, CR, and HR1 domains becomes bound to the membrane after ∼23 μs. The color code is as for Figure 6b. (ZIP)

## References

[ref1] Zhu N.; Zhang D.; Wang W.; Li X.; Yang B.; Song J.; Zhao X.; Huang B.; Shi W.; Lu R.; et al. A Novel Coronavirus from Patients with Pneumonia in China, 2019. N Engl J. Med. 2020, 382 (8), 727–733. 10.1056/NEJMoa2001017. 31978945 PMC7092803

[ref2] Tu Y. F.; Chien C. S.; Yarmishyn A. A.; Lin Y. Y.; Luo Y. H.; Lin Y. T.; Lai W. Y.; Yang D. M.; Chou S. J.; Yang Y. P.; et al. A Review of SARS-CoV-2 and the Ongoing Clinical Trials. Int. J. Mol. Sci. 2020, 21 (7), 2657 10.3390/ijms21072657. 32290293 PMC7177898

[ref3] Wrapp D.; Wang N.; Corbett K. S.; Goldsmith J. A.; Hsieh C. L.; Abiona O.; Graham B. S.; McLellan J. S. Cryo-EM structure of the 2019-nCoV spike in the prefusion conformation. Science 2020, 367 (6483), 1260–1263. 10.1126/science.abb2507. 32075877 PMC7164637

[ref4] Ziegler C. G. K.; Allon S. J.; Nyquist S. K.; Mbano I. M.; Miao V. N.; Tzouanas C. N.; Cao Y.; Yousif A. S.; Bals J.; Hauser B. M.; et al. SARS-CoV-2 Receptor ACE2 Is an Interferon-Stimulated Gene in Human Airway Epithelial Cells and Is Detected in Specific Cell Subsets across Tissues. Cell 2020, 181 (5), 1016–1035. 10.1016/j.cell.2020.04.035. 32413319 PMC7252096

[ref5] Harrison S. C. Viral membrane fusion. Nat. Struct Mol. Biol. 2008, 15 (7), 690–8. 10.1038/nsmb.1456. 18596815 PMC2517140

[ref6] Heald-Sargent T.; Gallagher T. Ready, set, fuse! The coronavirus spike protein and acquisition of fusion competence. Viruses 2012, 4 (4), 557–80. 10.3390/v4040557. 22590686 PMC3347323

[ref7] Cai Y.; Zhang J.; Xiao T.; Peng H.; Sterling S. M.; Walsh R. M. Jr; Rawson S.; Rits-Volloch S.; Chen B. Distinct conformational states of SARS-CoV-2 spike protein. Science 2020, 369 (6511), 1586–1592. 10.1126/science.abd4251. 32694201 PMC7464562

[ref8] Jaafar Z. A.; Kieft J. S. Viral RNA structure-based strategies to manipulate translation. Nat. Rev. Microbiol 2019, 17 (2), 110–123. 10.1038/s41579-018-0117-x. 30514982 PMC6452865

[ref9] Walls A. C.; Park Y. J.; Tortorici M. A.; Wall A.; McGuire A. T.; Veesler D. Structure, Function, and Antigenicity of the SARS-CoV-2 Spike Glycoprotein. Cell 2020, 181 (2), 281–292. 10.1016/j.cell.2020.02.058. 32155444 PMC7102599

[ref10] Xia S.; Liu M.; Wang C.; Xu W.; Lan Q.; Feng S.; Qi F.; Bao L.; Du L.; Liu S.; et al. Inhibition of SARS-CoV-2 (previously 2019-nCoV) infection by a highly potent pan-coronavirus fusion inhibitor targeting its spike protein that harbors a high capacity to mediate membrane fusion. Cell Res. 2020, 30 (4), 343–355. 10.1038/s41422-020-0305-x. 32231345 PMC7104723

[ref11] Ivanovic T.; Choi J. L.; Whelan S. P.; van Oijen A. M.; Harrison S. C. Influenza-virus membrane fusion by cooperative fold-back of stochastically induced hemagglutinin intermediates. Elife 2013, 2, e00333 10.7554/eLife.00333. 23550179 PMC3578201

[ref12] Ladinsky M. S.; Gnanapragasam P. N.; Yang Z.; West A. P.; Kay M. S.; Bjorkman P. J. Electron tomography visualization of HIV-1 fusion with target cells using fusion inhibitors to trap the pre-hairpin intermediate. Elife 2020, 9, e58411 10.7554/eLife.58411. 32697193 PMC7394545

[ref13] Benton D. J.; Gamblin S. J.; Rosenthal P. B.; Skehel J. J. Structural transitions in influenza haemagglutinin at membrane fusion pH. Nature 2020, 583 (7814), 150–153. 10.1038/s41586-020-2333-6. 32461688 PMC7116728

[ref14] Marcink T. C.; Wang T.; des Georges A.; Porotto M.; Moscona A. Human parainfluenza virus fusion complex glycoproteins imaged in action on authentic viral surfaces. PLoS Pathog 2020, 16 (9), e1008883 10.1371/journal.ppat.1008883. 32956394 PMC7529294

[ref15] Chen P.; Nirula A.; Heller B.; Gottlieb R. L.; Boscia J.; Morris J.; Huhn G.; Cardona J.; Mocherla B.; Stosor V.; et al. SARS-CoV-2 Neutralizing Antibody LY-CoV555 in Outpatients with Covid-19. N Engl J. Med. 2021, 384 (3), 229–237. 10.1056/NEJMoa2029849. 33113295 PMC7646625

[ref16] Baum A.; Ajithdoss D.; Copin R.; Zhou A.; Lanza K.; Negron N.; Ni M.; Wei Y.; Mohammadi K.; Musser B.; et al. REGN-COV2 antibodies prevent and treat SARS-CoV-2 infection in rhesus macaques and hamsters. Science 2020, 370 (6520), 1110–1115. 10.1126/science.abe2402. 33037066 PMC7857396

[ref17] Jackson L. A.; Anderson E. J.; Rouphael N. G.; Roberts P. C.; Makhene M.; Coler R. N.; McCullough M. P.; Chappell J. D.; Denison M. R.; Stevens L. J.; et al. An mRNA Vaccine against SARS-CoV-2 - Preliminary Report. N Engl J. Med. 2020, 383 (20), 1920–1931. 10.1056/NEJMoa2022483. 32663912 PMC7377258

[ref18] Walsh E. E.; Frenck R. W. Jr; Falsey A. R.; Kitchin N.; Absalon J.; Gurtman A.; Lockhart S.; Neuzil K.; Mulligan M. J.; Bailey R.; et al. Safety and Immunogenicity of Two RNA-Based Covid-19 Vaccine Candidates. N Engl J. Med. 2020, 383 (25), 2439–2450. 10.1056/NEJMoa2027906. 33053279 PMC7583697

[ref19] de Vries R. D.; Schmitz K. S.; Bovier F. T.; Predella C.; Khao J.; Noack D.; Haagmans B. L.; Herfst S.; Stearns K. N.; Drew-Bear J.; et al. Intranasal fusion inhibitory lipopeptide prevents direct-contact SARS-CoV-2 transmission in ferrets. Science 2021, 371 (6536), 1379–1382. 10.1126/science.abf4896. 33597220 PMC8011693

[ref20] Bosch B. J.; Martina B. E.; Van Der Zee R.; Lepault J.; Haijema B. J.; Versluis C.; Heck A. J.; De Groot R.; Osterhaus A. D.; Rottier P. J. Severe acute respiratory syndrome coronavirus (SARS-CoV) infection inhibition using spike protein heptad repeat-derived peptides. Proc. Natl. Acad. Sci. U. S. A. 2004, 101 (22), 8455–60. 10.1073/pnas.0400576101. 15150417 PMC420415

[ref21] Marcink T. C.; Yariv E.; Rybkina K.; Mas V.; Bovier F. T.; des Georges A.; Greninger A. L.; Alabi C. A.; Porotto M.; Ben-Tal N.; et al. Hijacking the Fusion Complex of Human Parainfluenza Virus as an Antiviral Strategy. mBio 2020, 11 (1), e03203-19 10.1128/mBio.03203-19. 32047132 PMC7018645

[ref22] LaBonte J.; Lebbos J.; Kirkpatrick P. Enfuvirtide. Nat. Rev. Drug Discov 2003, 2 (5), 345–6. 10.1038/nrd1091. 12755128

[ref23] Hoffmann M.; Arora P.; Gross R.; Seidel A.; Hornich B. F.; Hahn A. S.; Kruger N.; Graichen L.; Hofmann-Winkler H.; Kempf A.; et al. SARS-CoV-2 variants B.1.351 and P.1 escape from neutralizing antibodies. Cell 2021, 184 (9), 2384–2393. 10.1016/j.cell.2021.03.036. 33794143 PMC7980144

[ref24] Schmitz K. S.; Geers D.; de Vries R. D.; Bovier T. F.; Mykytyn A. Z.; Geurts van Kessel C. H.; Haagmans B. L.; Porotto M.; de Swart R. L.; Moscona A. Potency of Fusion-Inhibitory Lipopeptides against SARS-CoV-2 Variants of Concern. mBio 2022, 13 (3), e0124922 10.1128/mbio.01249-22. 35695453 PMC9239157

[ref25] Tang T.; Bidon M.; Jaimes J. A.; Whittaker G. R.; Daniel S. Coronavirus membrane fusion mechanism offers as a potential target for antiviral development. Antiviral Res. 2020, 178, 104792 10.1016/j.antiviral.2020.104792. 32272173 PMC7194977

[ref26] Casalino L.; Gaieb Z.; Goldsmith J. A.; Hjorth C. K.; Dommer A. C.; Harbison A. M.; Fogarty C. A.; Barros E. P.; Taylor B. C.; McLellan J. S.; et al. Beyond Shielding: The Roles of Glycans in the SARS-CoV-2 Spike Protein. ACS Cent Sci. 2020, 6 (10), 1722–1734. 10.1021/acscentsci.0c01056. 33140034 PMC7523240

[ref27] Turonova B.; Sikora M.; Schurmann C.; Hagen W. J. H.; Welsch S.; Blanc F. E. C.; von Bulow S.; Gecht M.; Bagola K.; Horner C.; et al. In situ structural analysis of SARS-CoV-2 spike reveals flexibility mediated by three hinges. Science 2020, 370 (6513), 203–208. 10.1126/science.abd5223. 32817270 PMC7665311

[ref28] Ali A.; Vijayan R. Dynamics of the ACE2-SARS-CoV-2/SARS-CoV spike protein interface reveal unique mechanisms. Sci. Rep 2020, 10 (1), 14214 10.1038/s41598-020-71188-3. 32848162 PMC7449962

[ref29] Khelashvili G.; Plante A.; Doktorova M.; Weinstein H. Ca(2+)-dependent mechanism of membrane insertion and destabilization by the SARS-CoV-2 fusion peptide. Biophys. J. 2021, 120 (6), 1105–1119. 10.1016/j.bpj.2021.02.023. 33631204 PMC7899928

[ref30] Gorgun D.; Lihan M.; Kapoor K.; Tajkhorshid E. Binding mode of SARS-CoV-2 fusion peptide to human cellular membrane. Biophys. J. 2021, 120 (14), 2914–2926. 10.1016/j.bpj.2021.02.041. 33675757 PMC7929786

[ref31] Borkotoky S.; Dey D.; Banerjee M. Computational Insight Into the Mechanism of SARS-CoV-2 Membrane Fusion. J. Chem. Inf Model 2021, 61 (1), 423–431. 10.1021/acs.jcim.0c01231. 33412850

[ref32] Yu A.; Pak A. J.; He P.; Monje-Galvan V.; Casalino L.; Gaieb Z.; Dommer A. C.; Amaro R. E.; Voth G. A. A multiscale coarse-grained model of the SARS-CoV-2 virion. Biophys. J. 2021, 120 (6), 1097–1104. 10.1016/j.bpj.2020.10.048. 33253634 PMC7695975

[ref33] Barfoot S.; Poger D.; Mark A. E. Understanding the Activated Form of a Class-I Fusion Protein: Modeling the Interaction of the Ebola Virus Glycoprotein 2 with a Lipid Bilayer. Biochemistry 2020, 59 (41), 4051–4058. 10.1021/acs.biochem.0c00527. 32960042

[ref34] Lin X.; Noel J. K.; Wang Q.; Ma J.; Onuchic J. N. Atomistic simulations indicate the functional loop-to-coiled-coil transition in influenza hemagglutinin is not downhill. Proc. Natl. Acad. Sci. U. S. A. 2018, 115 (34), E7905-E7913 10.1073/pnas.1805442115. 30012616 PMC6112712

[ref35] Lin M.; Da L. T. Refolding Dynamics of gp41 from Pre-fusion to Pre-hairpin States during HIV-1 Entry. J. Chem. Inf Model 2020, 60 (1), 162–174. 10.1021/acs.jcim.9b00746. 31845803

[ref36] Carr C. M.; Kim P. S. A spring-loaded mechanism for the conformational change of influenza hemagglutinin. Cell 1993, 73 (4), 823–32. 10.1016/0092-8674(93)90260-W. 8500173

[ref37] Hoffmann M.; Kleine-Weber H.; Pohlmann S. A Multibasic Cleavage Site in the Spike Protein of SARS-CoV-2 Is Essential for Infection of Human Lung Cells. Mol. Cell 2020, 78 (4), 779–784. 10.1016/j.molcel.2020.04.022. 32362314 PMC7194065

[ref38] Shang J.; Wan Y.; Luo C.; Ye G.; Geng Q.; Auerbach A.; Li F. Cell entry mechanisms of SARS-CoV-2. Proc. Natl. Acad. Sci. U. S. A. 2020, 117 (21), 11727–11734. 10.1073/pnas.2003138117. 32376634 PMC7260975

[ref39] Hoffmann M.; Kleine-Weber H.; Schroeder S.; Kruger N.; Herrler T.; Erichsen S.; Schiergens T. S.; Herrler G.; Wu N. H.; Nitsche A.; et al. SARS-CoV-2 Cell Entry Depends on ACE2 and TMPRSS2 and Is Blocked by a Clinically Proven Protease Inhibitor. Cell 2020, 181 (2), 271–280. 10.1016/j.cell.2020.02.052. 32142651 PMC7102627

[ref40] Benton D. J.; Wrobel A. G.; Xu P.; Roustan C.; Martin S. R.; Rosenthal P. B.; Skehel J. J.; Gamblin S. J. Receptor binding and priming of the spike protein of SARS-CoV-2 for membrane fusion. Nature 2020, 588 (7837), 327–330. 10.1038/s41586-020-2772-0. 32942285 PMC7116727

[ref41] Ou X.; Liu Y.; Lei X.; Li P.; Mi D.; Ren L.; Guo L.; Guo R.; Chen T.; Hu J.; et al. Characterization of spike glycoprotein of SARS-CoV-2 on virus entry and its immune cross-reactivity with SARS-CoV. Nat. Commun. 2020, 11 (1), 1620 10.1038/s41467-020-15562-9. 32221306 PMC7100515

[ref42] Hakansson-McReynolds S.; Jiang S.; Rong L.; Caffrey M. Solution structure of the severe acute respiratory syndrome-coronavirus heptad repeat 2 domain in the prefusion state. J. Biol. Chem. 2006, 281 (17), 11965–71. 10.1074/jbc.M601174200. 16507566 PMC8099417

[ref43] Xu D.; Zhang Y. Ab initio protein structure assembly using continuous structure fragments and optimized knowledge-based force field. Proteins 2012, 80 (7), 1715–35. 10.1002/prot.24065. 22411565 PMC3370074

[ref44] de Jong D. H.; Singh G.; Bennett W. F.; Arnarez C.; Wassenaar T. A.; Schafer L. V.; Periole X.; Tieleman D. P.; Marrink S. J. Improved Parameters for the Martini Coarse-Grained Protein Force Field. J. Chem. Theory Comput 2013, 9 (1), 687–97. 10.1021/ct300646g. 26589065

[ref45] Kabsch W.; Sander C. Dictionary of protein secondary structure: pattern recognition of hydrogen-bonded and geometrical features. Biopolymers 1983, 22 (12), 2577–637. 10.1002/bip.360221211. 6667333

[ref46] Poma A. B.; Cieplak M.; Theodorakis P. E. Combining the MARTINI and Structure-Based Coarse-Grained Approaches for the Molecular Dynamics Studies of Conformational Transitions in Proteins. J. Chem. Theory Comput. 2017, 13 (3), 1366–1374. 10.1021/acs.jctc.6b00986. 28195464

[ref47] Ke Z.; Oton J.; Qu K.; Cortese M.; Zila V.; McKeane L.; Nakane T.; Zivanov J.; Neufeldt C. J.; Cerikan B.; et al. Structures and distributions of SARS-CoV-2 spike proteins on intact virions. Nature 2020, 588 (7838), 498–502. 10.1038/s41586-020-2665-2. 32805734 PMC7116492

[ref48] Lai A. L.; Freed J. H. SARS-CoV-2 Fusion Peptide has a Greater Membrane Perturbating Effect than SARS-CoV with Highly Specific Dependence on Ca(2). J. Mol. Biol. 2021, 433 (10), 166946 10.1016/j.jmb.2021.166946. 33744314 PMC7969826

[ref49] O’Shaughnessy B.; Sawhney U. Polymer reaction kinetics at interfaces. Phys. Rev. Lett. 1996, 76 (18), 3444–3447. 10.1103/PhysRevLett.76.3444. 10060968

[ref50] Hills R. D. Jr; Lu L.; Voth G. A. Multiscale coarse-graining of the protein energy landscape. PLoS Comput. Biol. 2010, 6 (6), e1000827 10.1371/journal.pcbi.1000827. 20585614 PMC2891700

[ref51] Roel-Touris J.; Don C. G.; V. Honorato R.; Rodrigues J. P. G. L. M.; Bonvin A. M. J. J. Less Is More: Coarse-Grained Integrative Modeling of Large Biomolecular Assemblies with HADDOCK. J. Chem. Theory Comput 2019, 15 (11), 6358–6367. 10.1021/acs.jctc.9b00310. 31539250 PMC6854652

[ref52] Marrink S. J.; de Vries A. H.; Mark A. E. Coarse Grained Model for Semiquantitative Lipid Simulations. J. Phys. Chem. B 2004, 108 (2), 750–760. 10.1021/jp036508g.

[ref53] Marcink T. C.; Kicmal T.; Armbruster E.; Zhang Z.; Zipursky G.; Golub K. L.; Idris M.; Khao J.; Drew-Bear J.; McGill G.; et al. Intermediates in SARS-CoV-2 spike-mediated cell entry. Sci. Adv. 2022, 8 (33), eabo3153 10.1126/sciadv.abo3153. 35984891 PMC9390989

[ref54] Outlaw V. K.; Bovier F. T.; Mears M. C.; Cajimat M. N.; Zhu Y.; Lin M. J.; Addetia A.; Lieberman N. A. P.; Peddu V.; Xie X.; et al. Inhibition of Coronavirus Entry In Vitro and Ex Vivo by a Lipid-Conjugated Peptide Derived from the SARS-CoV-2 Spike Glycoprotein HRC Domain. mBio 2020, 11 (5), e01935-20 10.1128/mBio.01935-20. 33082259 PMC7587434

[ref55] Baylon J. L.; Tajkhorshid E. Capturing Spontaneous Membrane Insertion of the Influenza Virus Hemagglutinin Fusion Peptide. J. Phys. Chem. B 2015, 119 (25), 7882–93. 10.1021/acs.jpcb.5b02135. 25996559 PMC5040216

[ref56] Calder L. J.; Rosenthal P. B. Cryomicroscopy provides structural snapshots of influenza virus membrane fusion. Nat. Struct Mol. Biol. 2016, 23 (9), 853–8. 10.1038/nsmb.3271. 27501535 PMC6485592

[ref57] Benton D. J.; Nans A.; Calder L. J.; Turner J.; Neu U.; Lin Y. P.; Ketelaars E.; Kallewaard N. L.; Corti D.; Lanzavecchia A.; et al. Influenza hemagglutinin membrane anchor. Proc. Natl. Acad. Sci. U. S. A. 2018, 115 (40), 10112–10117. 10.1073/pnas.1810927115. 30224494 PMC6176637

[ref58] Lu L.; Liu Q.; Zhu Y.; Chan K. H.; Qin L.; Li Y.; Wang Q.; Chan J. F.; Du L.; Yu F.; et al. Structure-based discovery of Middle East respiratory syndrome coronavirus fusion inhibitor. Nat. Commun. 2014, 5 (1), 3067 10.1038/ncomms4067. 24473083 PMC7091805

[ref59] Wang C.; van Haperen R.; Gutierrez-Alvarez J.; Li W.; Okba N. M. A.; Albulescu I.; Widjaja I.; van Dieren B.; Fernandez-Delgado R.; Sola I.; et al. A conserved immunogenic and vulnerable site on the coronavirus spike protein delineated by cross-reactive monoclonal antibodies. Nat. Commun. 2021, 12 (1), 1715 10.1038/s41467-021-21968-w. 33731724 PMC7969777

[ref60] Webb B.; Sali A. Comparative Protein Structure Modeling Using MODELLER. Curr. Protoc Bioinformatics 2016, 54 (1), 5 6 1–5 6 37. 10.1002/cpbi.3. PMC5031415 27322406

[ref61] Bekker H.; Berendsen H.; Dijkstra E.; Achterop S.; Van Drunen R.; Van der Spoel D.; Sijbers A.; Keegstra H.; Reitsma B.; Renardus M. In Gromacs: A parallel computer for molecular dynamics simulations; Physics computing; World Scientific: Singapore, 1993; pp 252–256.

[ref62] Berendsen H. J. C.; van der Spoel D.; van Drunen R. GROMACS: A message-passing parallel molecular dynamics implementation. Comput. Phys. Commun. 1995, 91 (1–3), 43–56. 10.1016/0010-4655(95)00042-E.

[ref63] Jo S.; Kim T.; Iyer V. G.; Im W. CHARMM-GUI: a web-based graphical user interface for CHARMM. J. Comput. Chem. 2008, 29 (11), 1859–65. 10.1002/jcc.20945. 18351591

[ref64] Best R. B.; Zhu X.; Shim J.; Lopes P. E.; Mittal J.; Feig M.; MacKerell A. D. Jr Optimization of the additive CHARMM all-atom protein force field targeting improved sampling of the backbone ϕ, ψ and side-chain χ1 and χ2 dihedral angles. J. Chem. Theory Comput. 2012, 8 (9), 3257–3273. 10.1021/ct300400x. 23341755 PMC3549273

[ref65] Klauda J. B.; Venable R. M.; Freites J. A.; O’Connor J. W.; Tobias D. J.; Mondragon-Ramirez C.; Vorobyov I.; MacKerell A. D. Jr; Pastor R. W. Update of the CHARMM all-atom additive force field for lipids: validation on six lipid types. J. Phys. Chem. B 2010, 114 (23), 7830–43. 10.1021/jp101759q. 20496934 PMC2922408

[ref66] Joosten R. P.; te Beek T. A.; Krieger E.; Hekkelman M. L.; Hooft R. W.; Schneider R.; Sander C.; Vriend G. A series of PDB related databases for everyday needs. Nucleic Acids Res. 2011, 39, D411–9. 10.1093/nar/gkq1105. 21071423 PMC3013697

[ref67] Wassenaar T. A.; Pluhackova K.; Bockmann R. A.; Marrink S. J.; Tieleman D. P. Going Backward: A Flexible Geometric Approach to Reverse Transformation from Coarse Grained to Atomistic Models. J. Chem. Theory Comput 2014, 10 (2), 676–90. 10.1021/ct400617g. 26580045

[ref68] Marrink S. J.; Risselada H. J.; Yefimov S.; Tieleman D. P.; de Vries A. H. The MARTINI force field: coarse grained model for biomolecular simulations. J. Phys. Chem. B 2007, 111 (27), 7812–24. 10.1021/jp071097f. 17569554

[ref69] Wassenaar T. A.; Ingolfsson H. I.; Bockmann R. A.; Tieleman D. P.; Marrink S. J. Computational Lipidomics with insane: A Versatile Tool for Generating Custom Membranes for Molecular Simulations. J. Chem. Theory Comput 2015, 11 (5), 2144–55. 10.1021/acs.jctc.5b00209. 26574417

