# Supplementary material for: Host Cell Membrane Capture by the SARS-CoV-2 Spike Protein Fusion Intermediate
Source: ACS Cent Sci. 2023 Jun 7;9(6):1213–28. doi: 10.1021/acscentsci.3c00158 (PMC10255576; doi:10.1021/acscentsci.3c00158)
Supplement: Supplementary file 1 — oc3c00158_si_001.pdf [file oc3c00158_si_001.pdf]

Supporting Information

Host cell membrane capture by the SARS-CoV-2 spike protein fusion intermediate

Rui Su[1], Jin Zeng[1], Tara C. Marcink[2,3], Matteo Porotto[2,3,4], Anne Moscona[2,3,5,6] and Ben O'Shaughnessy[1,*]

[1]Department of Chemical Engineering, Columbia University, New York, NY, USA
[2]Department of Pediatrics, Columbia University Vagelos College of Physicians & Surgeons, New York, NY, USA
[3]Center for Host-Pathogen Interaction, Columbia University Vagelos College of Physicians & Surgeons, New York, NY, USA
[4]Department of Experimental Medicine, University of Campania "Luigi Vanvitelli", Naples, Italy
[5]Department of Microbiology & Immunology, Columbia University Vagelos College of Physicians & Surgeons, New York, NY, USA
[6]Department of Physiology & Columbia University Vagelos College of Physicians & Surgeons, New York, NY, USA

[*]Correspondence should be addressed to B.O'S (email: bo8@columbia.edu)

Table of contents

**Supplementary methods**	**S2-S6**
**Supplementary figures**	**S7-S19**
**Supplementary movies**	**S20**
**Supplementary references**	**S21**

Supplementary Methods

This section provides more detailed information for each simulation described in Methods.

All-atom simulation of the fusion intermediate.

The full-length fusion intermediate (FI, Fig. 1b) with its TMD inserted in the membrane was placed in a simulation box of $16 \times 16 \times 43\ \mathrm{nm}^3$. The membrane-protein system was built using the CHARMM-GUI membrane builder[1], consisting of 786 lipids for the pure DPPC membrane. The termini and ionizable residues were treated in their charged states assuming neutral pH. The disulfide bond in FP was added according to the prefusion structure (PDB: 6XR8), while the disulfide bonds in the other domains, BH and GP, were added based on the postfusion structure (PDB: 6XRA, the disulfide bonds were conserved in the pre- and postfusion structures). The resulting simulation box contained approximately 300,000 water molecules and was neutralized with 150 mM NaCl ions. The TIP3P model was used for water[2].

The system was first energy-minimized for 1,000 steps. Then, 2 equilibrations in the NVT ensemble were each performed for 0.1 ns with position restraints on all protein atoms. Subsequently, 4 equilibrations in the NPT ensembles were each performed for 0.5 ns with position restraints on protein heavy atoms. The production simulation was run for 406 ns in the NPT ensemble at 1 bar and 310 K and with a time step of 2 fs. The temperature and pressure were maintained using the Nose´-Hoover thermostat[3,4] and Parinello-Rahman barostat[5], respectively. All the energy-minimization, equilibration, and production simulations were performed using GROMACS 2019.6[6,7]. The secondary structure of each residue in the FI was analyzed using the *dssp* algorithm[8,9] for every 0.1 ns.

Coarse-grained simulation of a fusion intermediate with uncleaved S2′ sites.

Atomistic coordinates of the full-length fusion intermediate (Fig. 1b) were converted onto the MARTINI 2.2 topology using the *martinize* utility and placed in a simulation box of $40 \times 40 \times 50\ \mathrm{nm}^3$. The unsolved C-terminal part of the GP domain in the postfusion structure[10] (residues 771-815) was forced to be a loop, by changing the input secondary structural file to the *martinize* utility. The terminal and ionizable residues were treated in their charged states assuming neutral pH. The box was then solvated by approximately 600,000 coarse-grained water particles and was neutralized by 150 mM NaCl ions.

In each protomer, the FP N-terminus residue (residue 816) and the GP C-terminus residue (residue 815) were pulled together at a constant rate of 10 nm/μs by a harmonic potential with a force constant of $500\ \mathrm{kJ\ mol^{-1}\ nm^{-2}}$. Position constrains by a harmonic potential with force constant of $1{,}000\ \mathrm{kJ\ mol^{-1}\ nm^{-2}}$ were applied to the beads in the domains other than GP, FP and CR. The pulling simulation took ~1.5 μs so that in the final configuration, the COG distance between residues 815 and 816 in one of the three protomers reaches ~0.5 nm.

The final coordinates of the coarse-grained FI were then backmapped into atomistic resolution in CHARMM36 force field[11,12]. The residues 815 and 816 were covalently connected in the protomer with the smallest separation between the two residues, and this protomer was duplicated twice to make a trimer using Pymol. Now a structure of the FI with its S2′ sites uncleaved was created. Then, the all-atom structure of the FI with its S2′ sites uncleaved was converted into MARTINI coarse-grained representation using the *martinize* utility. A simulation box of $30 \times 30 \times 50\ \mathrm{nm}^3$ was generated using the *insane* utility, in which the coarse-grained FI with its S2′ sites uncleaved was inserted in a crystalline DPPC bilayer consisting of 2,831 coarse-grained lipids that represented the viral envelope.

The system containing the coarse-grained FI with its S2′ sites uncleaved on the viral envelope was first energy-minimized for 2,000 steps in the vacuum. Subsequently, the box was solvated with approximately 300,000 coarse-grained water particles and neutralized with 150 mM NaCl ions. The system was then energy-minimized for 2,000 steps and equilibrated for 4 ns in NPT ensemble sampling. Then the system was subjected to a production simulation lasting 4 μs in the NPT ensemble. The system temperature, membrane tension, and system pressure were maintained at the same value using the same thermostat and barostat as mentioned above.

Coarse-grained simulations of the fusion intermediate.

Atomistic coordinates of the modeled full length fusion intermediate (Fig. 2a) were converted onto the secondary structure based coarse-grained MARTINI 2.2 topology[13,14] using the *martinize* utility. The termini and ionizable residues were treated in their charged states assuming neutral pH. Disulfide bonds were added to the same residues as in the all-atom simulation. The simulation box of $30 \times 30 \times 50$ nm^3 was generated using the *insane* utility[15], in which the coarse-grained (CG) FI with its TMD inserted in a crystalline DPPC bilayer consisting of 3,024 coarse-grained lipids that represents the viral envelope.

The system was first energy-minimized for 2,000 steps in vacuum. Subsequently, the box was solvated with approximately 300,000 coarse-grained water particles and neutralized with 150 mM NaCl ions. The system was then energy-minimized for 2,000 steps and equilibrated for 4 ns in NPT ensemble sampling. Then the system was subjected to 5 independent production simulations, each lasting 8 μs in the NPT ensemble. Equations of motion were integrated using the Verlet leapfrog algorithm with a 80 fs time step. Bonds were constrained with the LINCS algorithm. The system temperature (310K) was maintained by the velocity rescale thermostat[16]. The membrane tension (0.05 pN/nm) and the system pressure (1 bar) were maintained by the Berendsen barostat with surface-tension coupling[17].

Fitting a curve to the fusion intermediate ectodomain

To fit a curve to represent the FI ectodomain backbone (residues 912-1237) in the MARTINI simulations of the FI (Figs. 3 and S2), we first represented the backbone by points. Each point represented a residue and its position was calculated by the averaged position of the MARTINI backbone bead (the non-sidechain bead) of each residue among the three protomers. The coordinates of the beads were extracted from the simulation trajectory using the *mdtraj*[18] python package. Then the FI backbone was divided into an upper part (residues 912-1191) and a lower part (1152-1237) with an overlapping region of 40 residues. Each part was separately smoothed by the following steps. (1) All points (x_i, y_i, z_i) were calibrated so that the center of gravity (COG) was at the origin, (2) All points were rotated so that the new x,y,z axis are aligned with the eigenvectors of the gyration tensor of the rotated points (x_i', y_i', z_i'). This rotation maximized the root mean square projected length onto the z axis. (3) A smoothed curve $(\overline{x_i'}, \overline{y_i'}, z_i')$ was generated by the LOWESS algorithm in python, in which the smoothed $\overline{x_i'}$ and $\overline{y_i'}$ value for each z_i' was set by its neighboring points spanning one tenth of the entire z range. Then the smoothed upper/lower part was rotated back to the original orientation and the overlapping region was averaged between the upper/lower parts. Finally the reconnected points were smoothed by the B-spline method with 4th order polynomial functions. To find the location (from the N- to the C-terminus) of each domain on the fitted the curve, the curve was first reparametrized by its normalized arclength. The location of each residue was determined as the normalized arclength of the nearest point on the fitted curve

to the position of this residue averaged over the three protomers, using only the backbone bead to locate the residues.

The isolated fusion peptide binding assay.

The atomistic structure of the fusion peptide (FP, residue 816-855) was extracted from the crystal structure of the prefusion structure (PDB: 6XR8). Then the atomistic structure of the FP was coarse-grained into the MARTINI representation. The ionizable residues were treated in their charged states assuming neutral pH. The C-terminal carboxyl group at residue 855 was neutralized by changing the type of the backbone bead from Qa to Na and changing the backbone bead charge from -1 to 0 in the itp topology file, as the residue 855 connects CR in the full-length FI. The disulfide bond was added according to the solved prefusion structure (PDB: 6XR8). The coarse-grained FP was placed approximately 1 nm above a crystalline DPPC bilayer consisting of 162 coarse-grained lipids in a $7 \times 7 \times 10\,\mathrm{nm}^3$ box using the *insane* utility[15]. By implementing periodic boundary condition, this is equivalent to placing a FP between two planar membranes separated by ~5.5 nm.The system was first energy-minimized for 500 steps in vacuum. Subsequently, the box was solvated with approximately 2,000 coarse-grained water particles and neutralized with 150 mM NaCl ions. The system was then energy-minimized for 500 steps and equilibrated for 4 ns in the NPT ensemble with a 80 fs time step. Then the system was subjected to 10 independent production simulations, each lasting 24 μs in the NPT ensemble. The system temperature (310K) was maintained by the velocity rescale thermostat[16]. The membrane tension (0.05 pN/nm) and the system pressure (1 bar) were maintained by the Berendsen barostat with surface-tension coupling[17]. A binding event was defined to be when the z coordinate of the FP COG first had a value that positioned it below the upper membrane leaflet and above the lower membrane leaflet, where the leaflet locations were defined as the average locations of the PO4 beads in each leaflet.

All-atom simulation of a membrane-bound fusion peptide.

In one of the CG simulations in the FP-only binding assay, the final configuration of the FP and the membrane to which it was bound is converted to atomic resolution in CHARMM36 force field[11,12] using the *backward* utility[19]. The ionizable residues are treated in their charged states assuming neutral pH. The C-terminal carboxyl group is neutralized. The Disulfide bond is added to the same residues as in the FP-only binding assay. The resulting simulation box contained approximately 9,000 water molecules and was neutralized with 150 mM NaCl ions. The TIP3P model was used for water[2].

4 equilibrations in the NVT and NPT ensembles were each performed for 50 ns with position restraints on peptide heavy atoms. The production simulation was run for 2 μs in NPT ensembles at 1 bar and 310 K and with a time step of 2 fs. The temperature and pressure were maintained using the Nose´-Hoover thermostat[3,4] and Parinello-Rahman barostat with isotropic coupling[5], respectively. The secondary structure of each residue in the FP was analyzed using the *dssp* algorithm[8,9] for every 0.1 ns. The membrane insertion depth of each FP residue was defined as the vertical distance between the residue COG and the COG of PO_4 groups in the membrane leaflet to which the FP was bound.

Coarse-grained simulations of an equilibrated fusion peptide bound to a membrane.

In the AA simulation of a membrane-bounded FP simulation, the final configuration of the FP and the membrane to which it was bound was converted to MARTINI CG representation using the *martinize* utility. The ionizable residues were treated in their charged state. The C-terminal carboxyl group was neutralized.

The system was first energy-minimized for 1,000 steps in the vacuum. Subsequently, the box was solvated with water particles and ions to attain a salt concentration of 0.15 M. The system was then energy-minimized for 5,000 steps and equilibrated for 0.8 ns in the NPT ensemble. Then the system was subjected to the production simulation for 80 μs in the NPT ensemble. The system temperature (310K) and pressure (1 bar) were maintained by the velocity rescale thermostat and Parinello-Rahman barostat, respectively.

The membrane insertion depth of each residue was defined in the same way as for the AA simulation (see above). The gyration tensor M of the FP was defined as

$$M = \frac{1}{N}\sum_{\mathrm{i}=1}^{N}(r_{\mathrm{i}} - r_{\mathrm{c}})\otimes(r_{\mathrm{i}} - r_{\mathrm{c}}),$$

where $\otimes$ represents the dyadic product, r_{i} is the coordinate of the ith bead in the FP, N is the total number of beads, and $\mathrm{r_c}$ is the COG of the FP, as $\mathrm{r_c} = \sum_{i=1}^{N} \mathrm{r}_i\,/N$. The radius of gyration R_{g} was computed as the square root of the trace of M. The length and the width of the FP were defined as, respectively, greater and smaller of the eigenvalues of M projected onto the x-y plane, and the thickness was defined as $\sqrt{M_{\mathrm{zz}}}$.

Coarse-grained simulations of the fusion intermediate interacting with a target membrane. For ten of the simulations of the FI interacting with a target membrane, ten snapshots from three FI simulations (one membrane) were chosen as the initial condition in which the FI ectodomain protruded ~20 nm normal to the membrane. Another pre-equilibrated planar membrane (run for 4 μs) was placed 20 nm above the membrane anchoring the FI in the selected configurations. Each configuration containing an FI and two membranes was re-solvated by approximately 200,000 coarse-grained water particles and neutralized by 150 mM NaCl ions. Then, each of the 10 systems was equilibrated for 4 ns, and subject to a production simulation for 8 μs in the NPT ensemble. The system temperature (310K) was maintained by the velocity rescale thermostat. The membrane tension (0.05 pN/nm) of the two membranes and the system pressure (1 bar) was maintained by the Berendsen barostat with surface-tension coupling.

In an additional set of simulations, all ten runs started from a biased initial condition, in which the COG of the nearest N-terminal FP helix was within 1 nm of the adjacent membrane and the FI head was in the (1,2) configuration. Here, the membrane position was defined to be the mean location of all the PO4 beads in the lower leaflet of the upper membrane. Each production simulation lasted 22.4 μs with the same system temperature, pressure and the membrane tension.

Coarse-grained simulations of membrane binding by the partial fusion intermediate. The structure of the HR1, CR and FP domains in the MARTINI CG representation was extracted from the final configuration of a simulation of the FI interacting with a target membrane. The ionizable residues were treated in their charged states assuming neutral pH. The C-terminal carboxyl group at residue 984 was neutralized by changing the type of the backbone bead from Qa to Na and changing the backbone bead charge from -1 to 0 in the itp topology file, as the residue 984 connects CH in the full-length FI. This partial FI was positioned above ~ 0.5 nm above a crystalline DPPC bilayer consisting of 676 coarse-grained lipids in a 20 × 20 × 13 nm^3 box with periodic boundary conditions, equivalent to two planar membranes separated by ~8.3 nm.

The system was first energy-minimized for 500 steps in vacuum. Subsequently, the box was solvated with approximately 26,000 coarse-grained water particles and neutralized with 150 mM NaCl ions. The system was then energy-minimized for 2,000 steps and equilibrated for 4 ns in the NPT ensemble with a 80 fs time step. Then the system was subjected to 6 independent production simulations, each lasting 160 μs in the NPT ensemble. The system temperature (310K) was maintained by the velocity rescale thermostat[16]. The membrane tension (0.05 pN/nm) and the system pressure (1 bar) were maintained by the Berendsen barostat with surface-tension coupling[17].

The exact time for a binding event was defined to be when the vertical distance between the FP N-terminal helix COG and the membrane first had a value smaller than the averaged FP-membrane distance when the partial FI was stably bound. The membrane locations were defined as the average locations of the PO4 beads in leaflet to which the partial FI was bound.

To infer the averaged binding time τ of a partial FI, we assumed that at time t the unbound probability P follows an exponential function $P = \exp(-t/\tau)$. Given that at the end of the six simulations $t = 160\ \mu s$ the unbound probability is $4/6$, we estimated $\tau = 390 \pm 280\ \mu s$, where the uncertainty of the estimation is obtained using from error propagation.

Pulling the fusion peptide into the target membrane.

An initial condition was chosen with the nearest N-terminal FP helix lying within 1 nm of the membrane and with the FI head in the (1,2) configuration. The FP N-terminal helix was pulled vertically towards the upper membrane at a constant rate of 2.5 nm/μs by a harmonic potential with a force constant of $1{,}000\ \mathrm{kJ\ mol^{-1}\ nm^{-2}}$. The system temperature, membrane tension, and system pressure were maintained at the same value using the same thermostat and barostat as mentioned above. The pulling process took ~1.2 μs. In the final configuration the distance between the COG of the FP N-terminal helix and the COG of the entire upper membrane reached ~0.1 nm, and the FP N-terminal helix was pulled into the adjacent membrane patch by ~1 nm. Then the force was released and a subsequent 8 μs CG simulation was run, in which the system temperature, membrane tension, and system pressure were maintained at the same value using the same thermostat and barostat.

Supplementary Figures

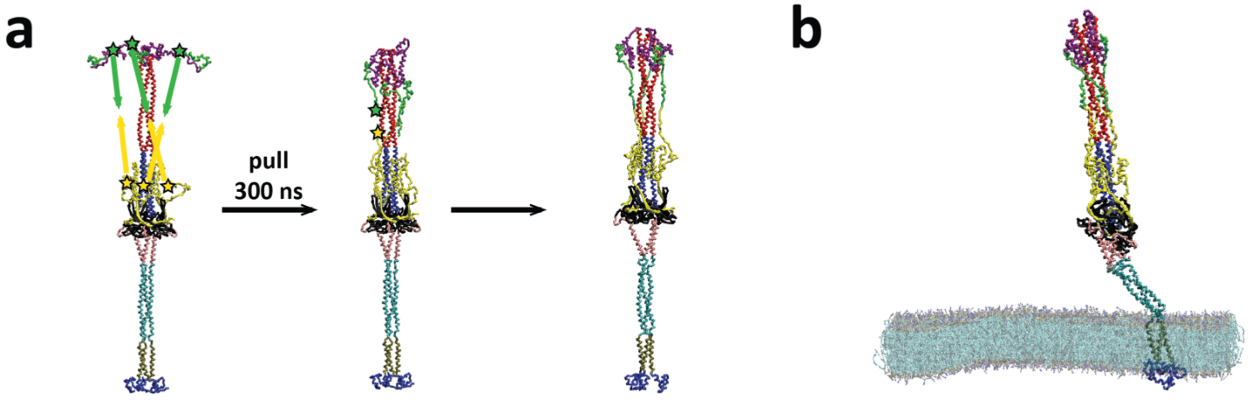

Supplementary Figure 1. Simulation of the fusion intermediate with uncleaved S2′ sites.
(a) Construction procedure for a fusion intermediate with uncleaved S2′ sites. Starting from the model structure in Fig. 2b, the C-terminus of three GPs and the N-terminus of three FPs were pulled together in ~1.5 μs in a MARTINI CG simulation. Then the C-terminus of GP and the N-terminus of FP in one protomer were connected covalently (stars). This protomer was duplicated twice to generate a FI homotrimer with uncleaved S2′ sites.
(b) Snapshot from a 4 μs simulation. The fusion peptides (green) are sequestered. The FI with uncleaved S2′ sites exhibits similar flexibility to the normally cleaved FI.

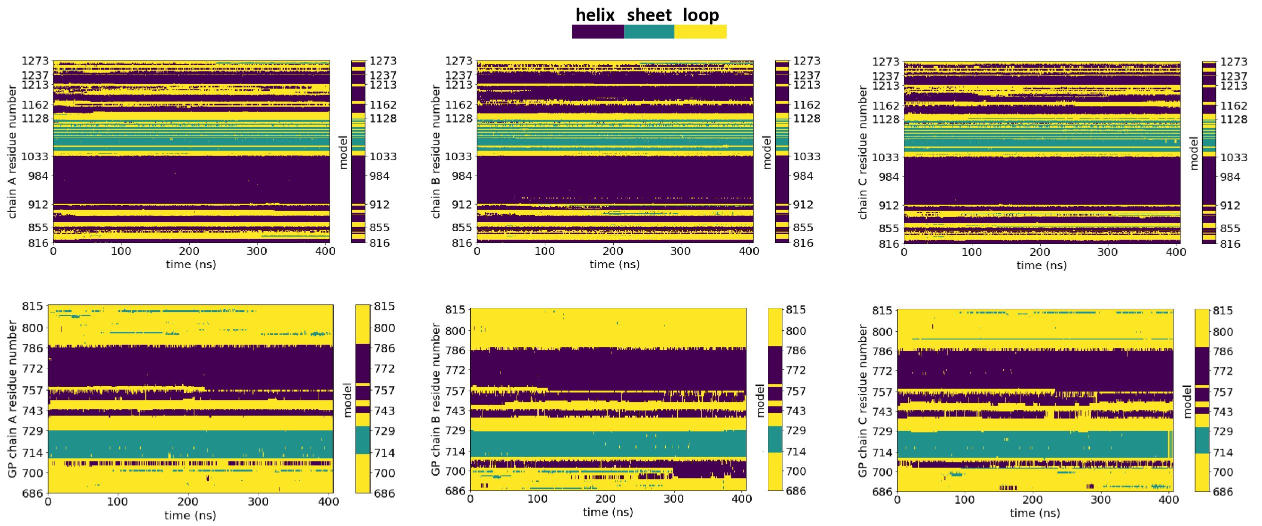

Supplementary Figure 2. Evolution of the secondary structure of the fusion intermediate during 406 ns of all-atom simulation.

The secondary structure of each residue was measured every 0.1 ns during the AA simulation (Fig. 2). Each panel refers to one protomer of either the main body of the S2 subunit (top row) or the cleaved GP (bottom row). The secondary structure of the fusion intermediate model of Figure 1b is shown to the right of each panel for comparison.

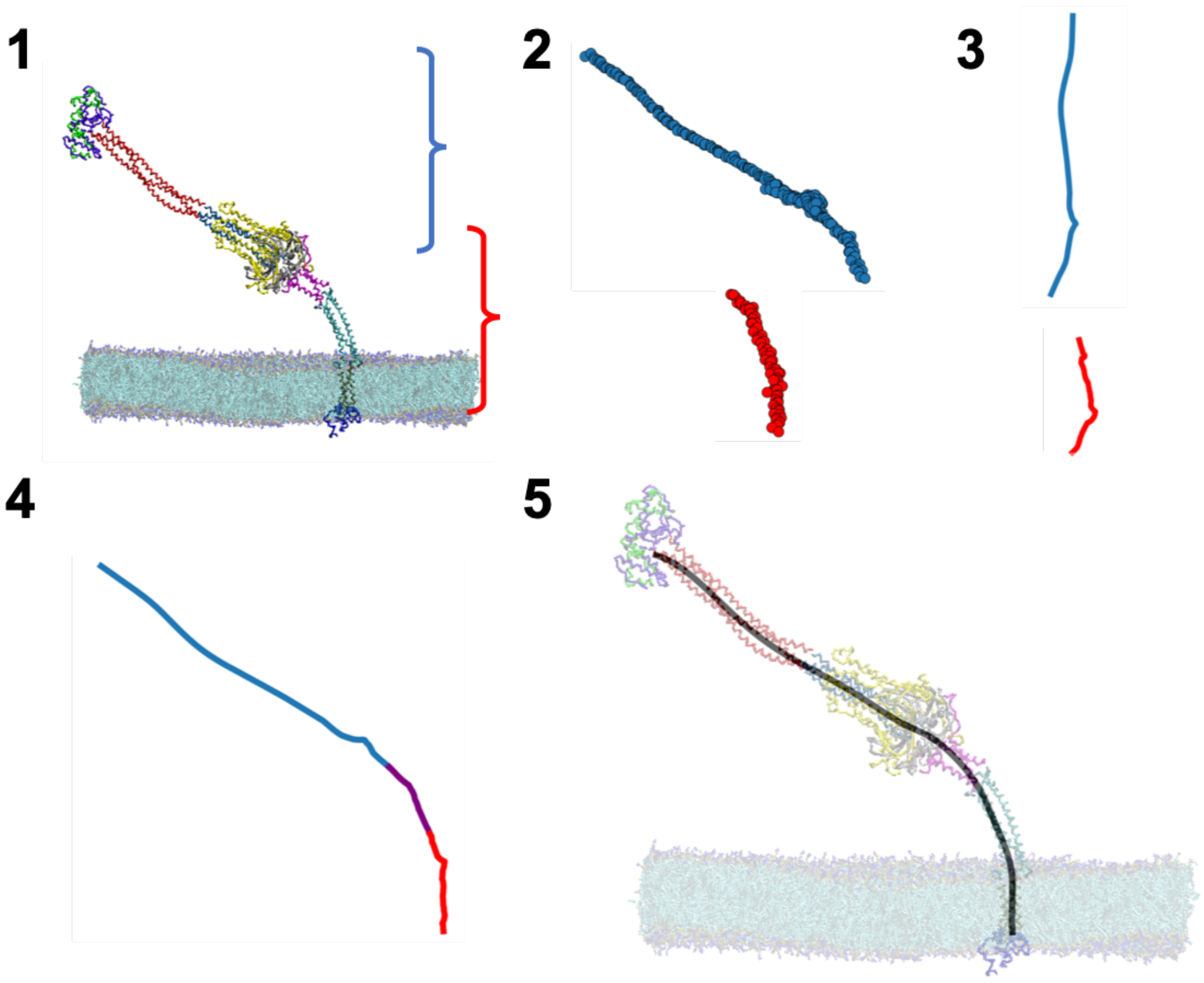

Supplementary Figure 3. Fitting a curve to represent the fusion intermediate ectodomain. (1) The coordinate of each coarse-grained bead is extracted by Python package *mdtraj*. (2) Each residue is represented by one point, averaged over three protomers. The upper part of the FI (blue, residues 912-1191) and the lower part (red, residues 1152-1237) are separated. (3) The upper and the lower parts are rotated so that the three principal axes are aligned with three eigenvectors of the gyration tensor of the rotated points. The rotated points are smoothed by the LOWESS algorithm. (4) The upper and lower parts are rotated back to their original orientations and reconnected. The overlapping region (purple, residues 1152-1191) is averaged over the upper and lower parts. (5) The final curve is fitted by the B-spline method.

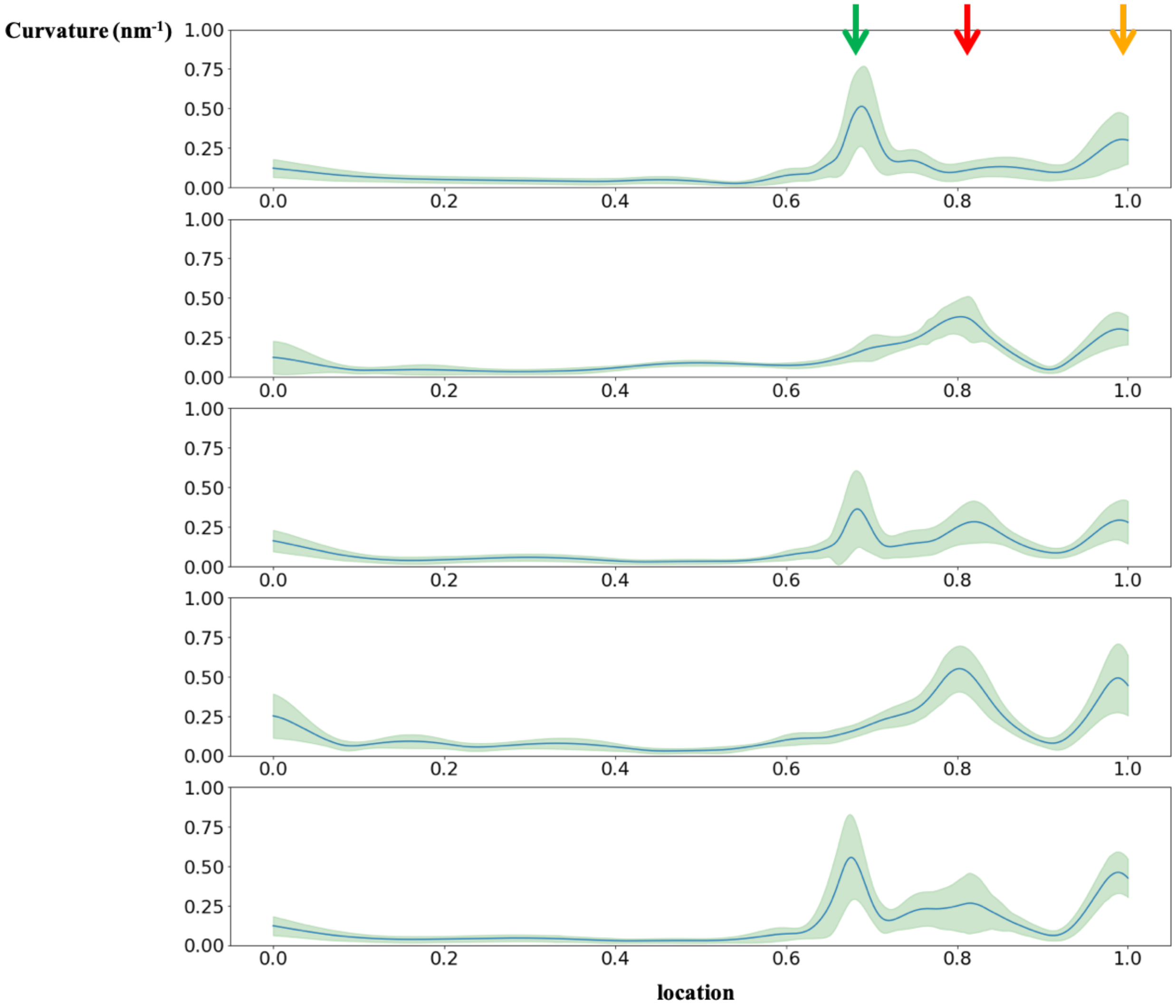

Supplementary Figure 4. Time averaged backbone curvature versus normalized backbone arclength for five parallel runs. The three hinges (Fig. 3b, arrows) show different magnitudes of curvature in the five parallel runs. Green envelope indicates SD.

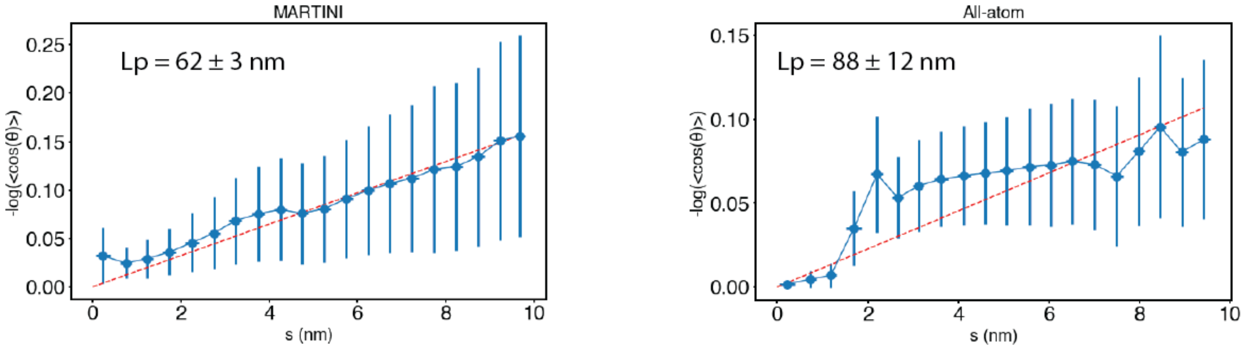

Supplementary Figure 5. Persistence length of the HR1 domain of the fusion intermediate measured in MARTINI coarse-grained and all-atom simulations. We used a curve to represent the orientation of the HR1 coiled-coil. In this curve, each residue is represented by one point, averaged over three protomers. At each point along this curve, we measured the contour length s and the angle difference $\theta(s)$ between the tangent line at this point and the tangent line at $s = 0$. The persistence length L_{P} has the relation with θ and s such that $\log < \cos\theta > = -s/L_{\mathrm{P}}$, where $< \cdots >$ indicates averaging over the 40 μs trajectory for the MARTINI simulation and 0.4 μs for the all-atom simulation. Thus, to calculate L_{P} we fitted a straight line in the plot of $-\log < \cos\theta >$ vs. s and the inverse of the slope is the persistence length L_{P}.

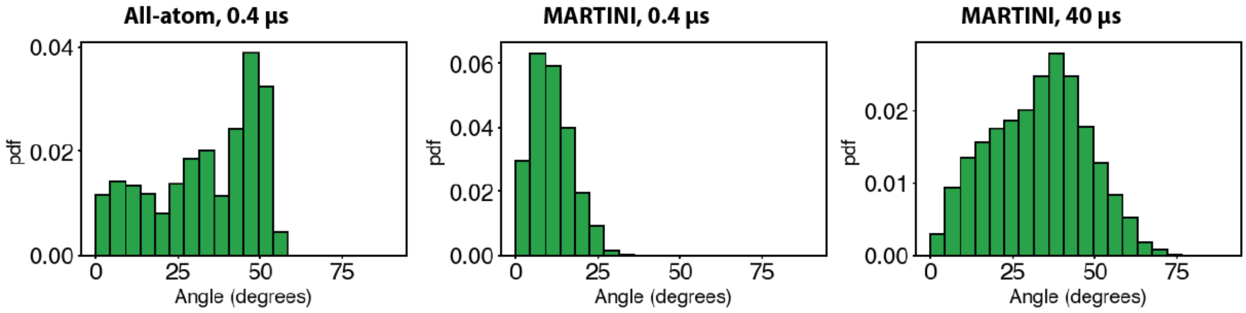

Supplementary Figure 6. Orientation angles of the fusion intermediate measured in all-atom and MARTINI coarse-grained simulations. We measured the orientation angle of the fusion intermediate in all-atom simulations using the same method as for Figure 3d. For a total 0.4 μs of simulation time, the fusion intermediate reached a larger range of orientation angles in all-atom simulations (left) than in MARTINI simulations (middle), possibly due to the more dynamical secondary structure in the base. However, for a total 40 μs of CG simulations, the FI reached the same range of angles as the 0.4 μs AA simulations.

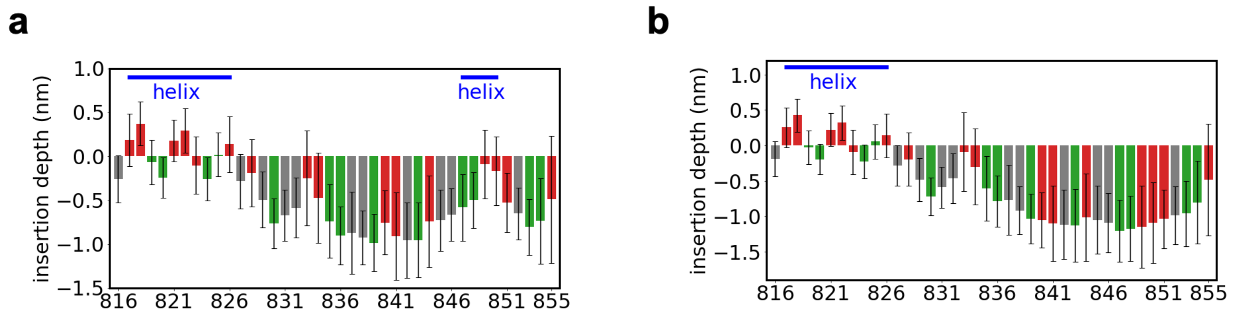

Supplementary Figure 7. Fusion peptide residue insertion depth measured in MARTINI coarse-grained simulations.

The insertion depth is defined as the vertical distance between the center of gravity (COG) of each fusion peptide residue and the COG of the PO_4 beads in the leaflet to which the fusion peptide is bound (same definition as for the all-atom simulation). The secondary structure used is **(a)** the equilibrated secondary structure from the all-atom simulation, and **(b)** the equilibrated secondary structure but imposing the C-terminal helix to be a loop. The insertion depths are averaged over the last 78 μs. Error bars: SD over the same time frame. Bars were colored by the hydrophobicity of the corresponding residues (red: hydrophobic, grey: neutral, blue: hydrophilic), using the same color scheme as in Fig. 4b.

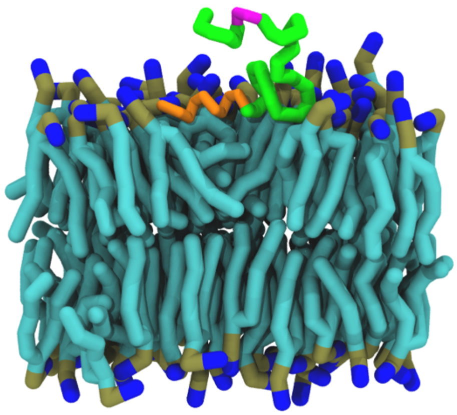

Supplementary Figure 8. MARTINI simulation snapshot showing a transient detachment of the fusion peptide C-terminal helix
The fusion peptide C-terminal helix (purple) unanchored transiently from the membrane for ~0.3 μs (the instance depicted here occurred after ~52 μs of the simulation). In contrast, the N-terminal helix (orange) always remained buried in the membrane.

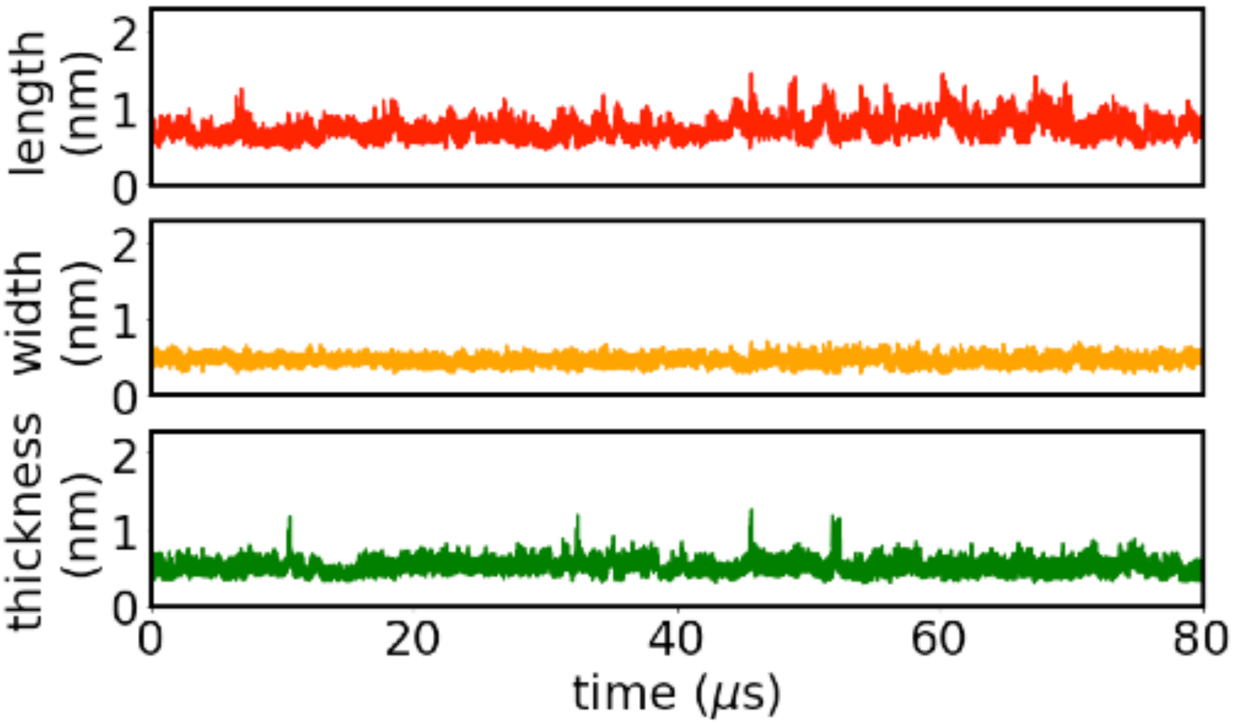

Supplementary Figure 9. Measured length, width and thickness versus time of the fusion peptide during the MARTINI coarse-grained simulation of an equilibrated fusion peptide bound to a membrane.

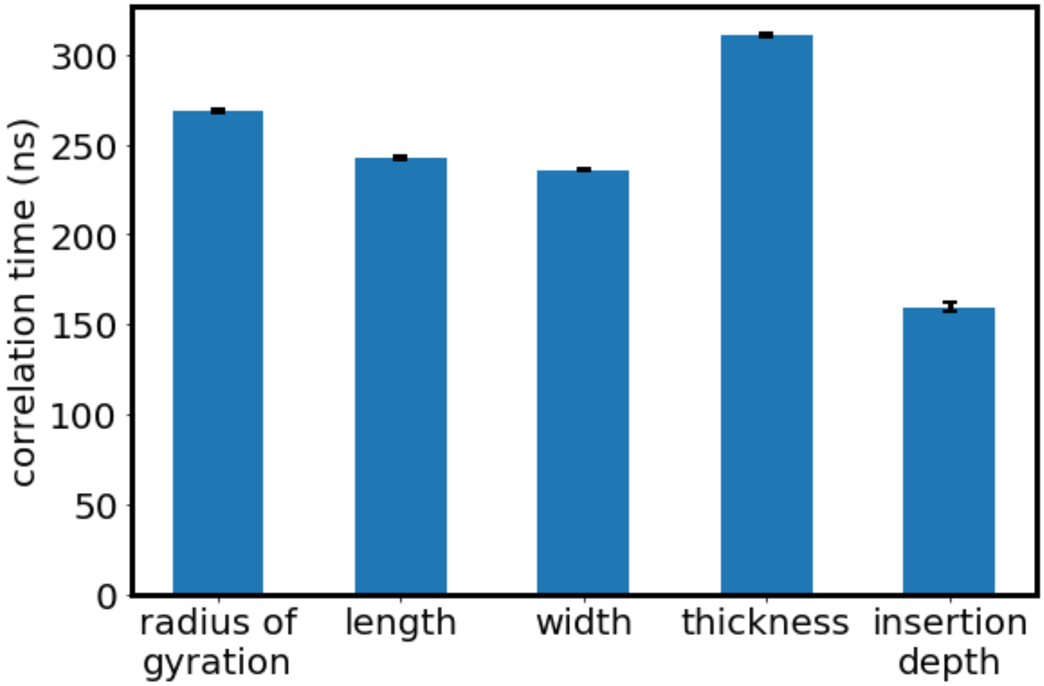

Supplementary Figure 10. Correlation times of the fusion peptide shape properties during MARTINI coarse-grained simulation of an equilibrated fusion peptide bound to a membrane.
The radius of gyration, length, width, thickness and insertion depth of the fusion peptide are calculated for the last 78 µs of the MARTINI simulation. The radius of gyration, length, width and thickness are calculated from the gyration tensor (see “Methods”). The insertion depth of the entire fusion peptide is defined as the vertical distance between the center of gravity of the fusion peptide and the center of gravity of the PO_4 beads in the leaflet to which the FP is bound. The correlation time is calculated in the same way as in Fig. 4g. Error bars: 95% confidence interval.

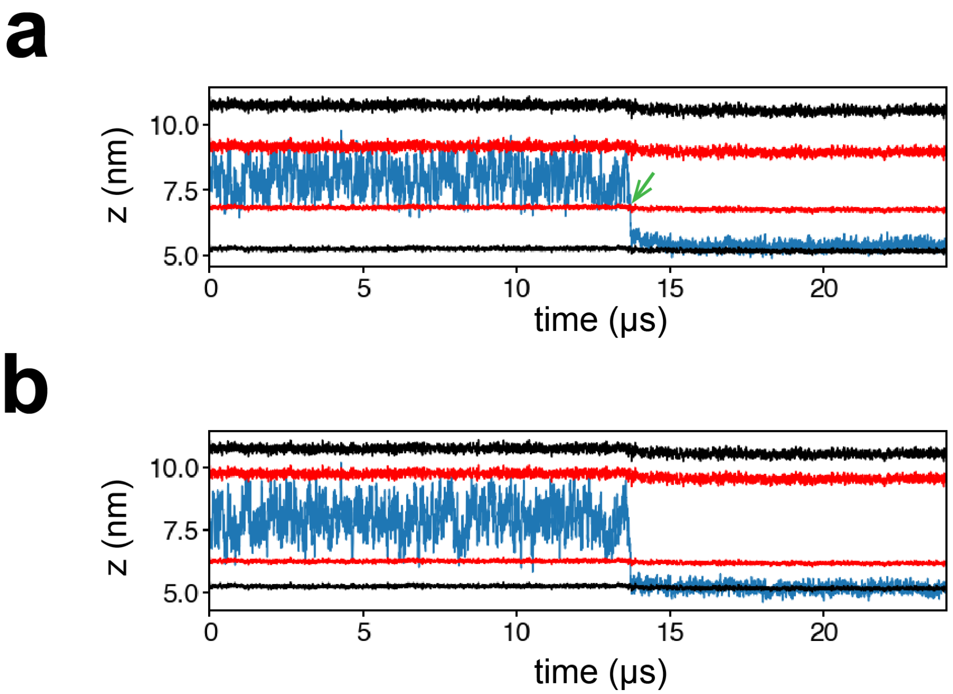

Supplementary Figure 11. Evolution of fusion peptide vertical position during a binding assay of an isolated fusion peptide.

(a) Evolution of the center of gravity of the fusion peptide (blue). The vertical positions of the two leaflets to which the FP is likely to bind are calculated by averaging the PO_4 bead positions in each leaflet (black). A collision event is defined as an approach to the membrane to within $R_{\mathrm{FP}} \sim 1.6$ nm of either leaflet (black), where R_{FP} is the rms FP end-to-end distance. A binding event is defined to be when the FP center of gravity first has a value that positioned it below the upper membrane leaflet and above the lower membrane leaflet (green arrow).

(b) Evolution of the center of gravity of the fusion peptide N-terminal helix (blue). The positions of the two leaflets are defined in the same way as for (a). Before the binding event, the N-terminal helix approached several times to within 1 nm of either leaflet (red).

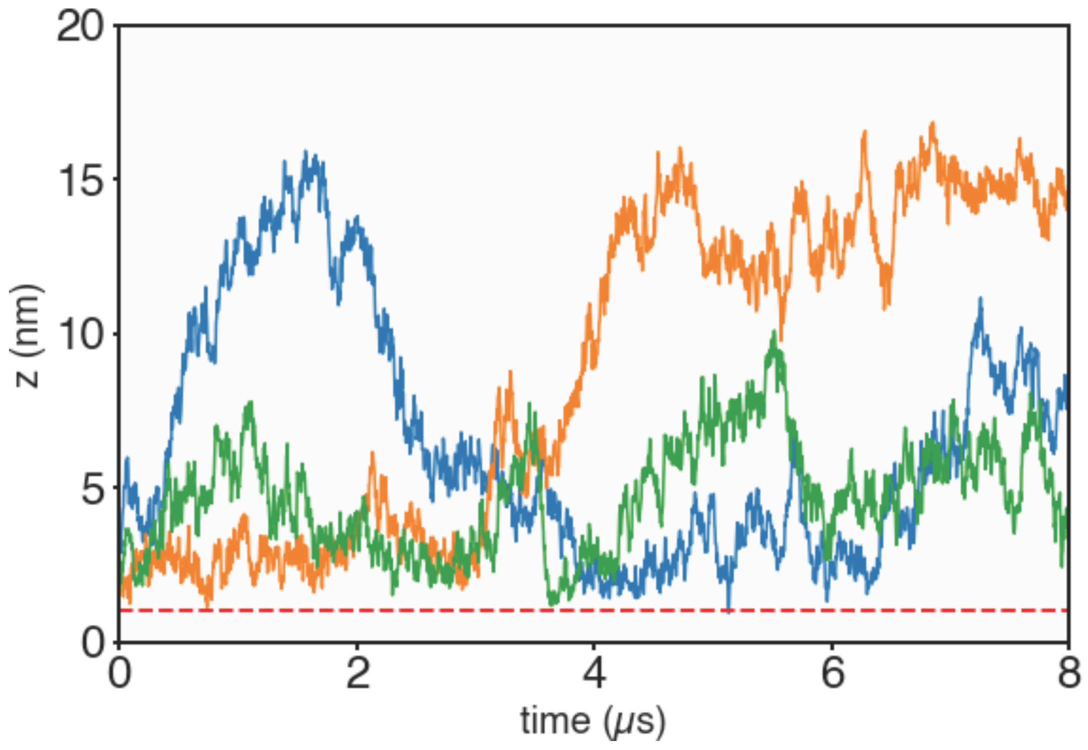

Supplementary Figure 12. Distance of the nearest FP N-terminal helix from the membrane versus time in three runs. Here the position of the nearest FP N-terminal helix was defined to be its COG location. The membrane position was defined to be the mean location of all the PO_4 beads in the lower leaflet of the upper membrane.

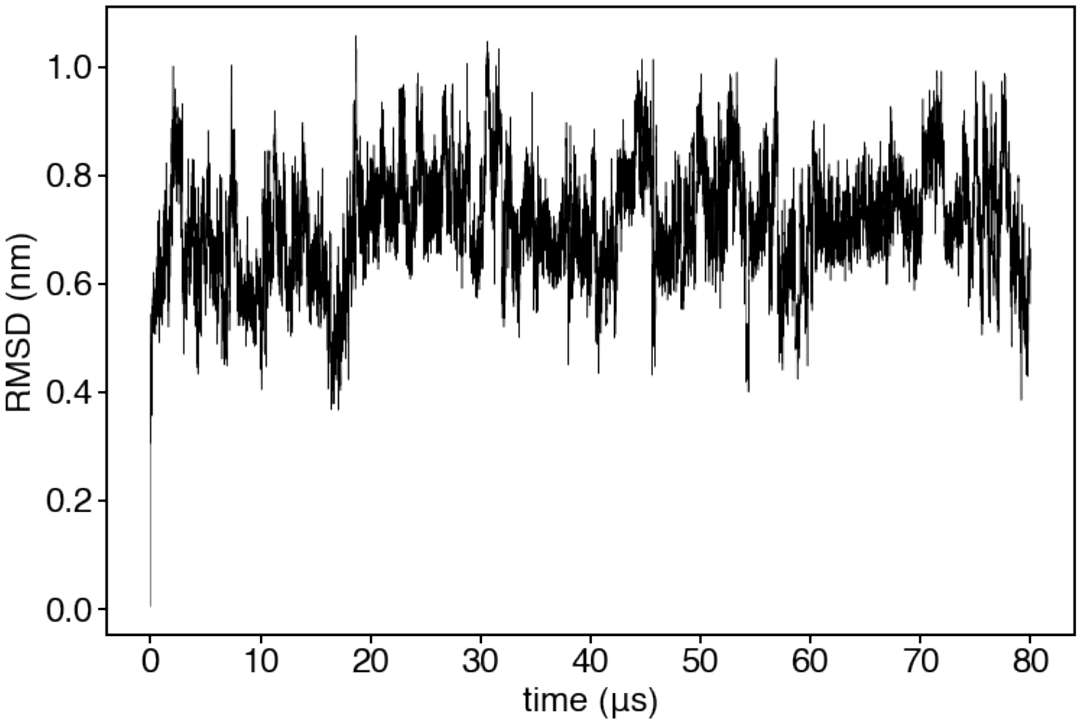

Supplementary Figure 13. Root-mean-square deviation (RMSD) of the locations of the backbone beads from their initial locations for a membrane-bound fusion peptide (Fig. 4). The RMSD is shown as a function of time for the same MARTINI simulation trajectory as that whose properties are shown in Figure 4f. The time-averaged RMSD is ~0.7 nm.

Supplementary Movies

Supplementary Movie 1. All-atom simulation of the SARS-CoV-2 fusion intermediate. The color code is as for Fig. 2.

Supplementary Movie 2. Coarse-grained MARTINI simulation of the SARS-CoV-2 fusion intermediate. The three hinges are colored according to the code of Fig. 3c.

Supplementary Movie 3. Simulation of Movie 2 with the fitted curve. A fitted curve (red) to the ectodomain backbone of the fusion intermediate (blue) whose TMD is anchored in the viral envelope (green).

Supplementary Movie 4. All-atom simulation of a membrane-bound fusion peptide (side view). The residues are colored according to their hydrophobicity, using the color scheme of Fig. 4b.

Supplementary Movie 5. Simulation of Movie 4, top view.

Supplementary Movie 6. Coarse-grained MARTINI simulation of an equilibrated fusion peptide bound to a membrane (side view). The N-terminal helix (orange) was always buried in the membrane, but after ~52 μs of the simulation the C-terminal helix (purple) transiently unanchored from the membrane for ~0.3 μs.

Supplementary Movie 7. Simulation of Movie 6, top view.

Supplementary Movie 8. Binding assay for an isolated fusion peptide (coarse-grained MARTINI simulation). The fusion peptide, removed from the FI, becomes bound to the membrane after ~1.6 μs, with the N-terminal helix (orange) providing first stable contact. The fusion peptide remains bound for the remaining ~22.4 μs of the simulation. The binding events of Fig. 5b are snapshots from this movie.

Supplementary Movie 9. Simulation of the fusion intermediate interacting with a target membrane, 8 μs duration. One of the fusion peptide N-terminal helices (green spheres) repeatedly approaches the membrane to within 1 nm, but fails to bind. Fig. 6e shows a snapshot from this movie.

Supplementary Movie 10. Binding assay of a partial fusion intermediate (coarse-grained MARTINI simulation, first 50 μs). The partial FI consisting of the FP, CR and HR1 domains becomes bound to the membrane after ~23 μs. The color code is as for Fig. 6b.

Supplementary References

1 Jo, S., Kim, T., Iyer, V. G. & Im, W. J. J. o. c. c. CHARMM-GUI: a web-based graphical user interface for CHARMM. **29**, 1859-1865 (2008).

2 Jorgensen, W. L., Chandrasekhar, J., Madura, J. D., Impey, R. W. & Klein, M. L. J. T. J. o. c. p. Comparison of simple potential functions for simulating liquid water. **79**, 926-935 (1983).

3 Hoover, W. G. J. P. r. A. Canonical dynamics: Equilibrium phase-space distributions. **31**, 1695 (1985).

4 Nosé, S. J. T. J. o. c. p. A unified formulation of the constant temperature molecular dynamics methods. **81**, 511-519 (1984).

5 Parrinello, M. & Rahman, A. J. J. o. A. p. Polymorphic transitions in single crystals: A new molecular dynamics method. **52**, 7182-7190 (1981).

6 Bekker, H. *et al.* in *Physics computing.* 252-256 (World Scientific Singapore).

7 Berendsen, H. J., van der Spoel, D. & van Drunen, R. J. C. p. c. GROMACS: a message-passing parallel molecular dynamics implementation. **91**, 43-56 (1995).

8 Kabsch, W. & Sander, C. J. B. O. R. o. B. Dictionary of protein secondary structure: pattern recognition of hydrogen-bonded and geometrical features. **22**, 2577-2637 (1983).

9 Joosten, R. P. *et al.* A series of PDB related databases for everyday needs. **39**, D411-D419 (2010).

10 Cai, Y. *et al.* Distinct conformational states of SARS-CoV-2 spike protein. **369**, 1586-1592 (2020).

11 Best, R. B. *et al.* Optimization of the additive CHARMM all-atom protein force field targeting improved sampling of the backbone φ, ψ and side-chain χ1 and χ2 dihedral angles. **8**, 3257-3273 (2012).

12 Klauda, J. B. *et al.* Update of the CHARMM all-atom additive force field for lipids: validation on six lipid types. **114**, 7830-7843 (2010).

13 de Jong, D. H. *et al.* Improved parameters for the martini coarse-grained protein force field. **9**, 687-697 (2013).

14 Marrink, S. J., Risselada, H. J., Yefimov, S., Tieleman, D. P. & De Vries, A. H. J. T. j. o. p. c. B. The MARTINI force field: coarse grained model for biomolecular simulations. **111**, 7812-7824 (2007).

15 Wassenaar, T. A. *et al.* Computational lipidomics with insane: a versatile tool for generating custom membranes for molecular simulations. **11**, 2144-2155 (2015).

16 Bussi, G., Donadio, D. & Parrinello, M. J. T. J. o. c. p. Canonical sampling through velocity rescaling. **126**, 014101 (2007).

17 Berendsen, H. J., Postma, J. v., van Gunsteren, W. F., DiNola, A. & Haak, J. R. J. T. J. o. c. p. Molecular dynamics with coupling to an external bath. **81**, 3684-3690 (1984).

18 McGibbon, R. T. *et al.* MDTraj: a modern open library for the analysis of molecular dynamics trajectories. **109**, 1528-1532 (2015).

19 Wassenaar, T. A. *et al.* Going backward: a flexible geometric approach to reverse transformation from coarse grained to atomistic models. **10**, 676-690 (2014).
